# Supplementary material for: Antimicrobial stewardship: Attitudes and practices of healthcare providers in selected health facilities in Uganda
Source: PLoS One. 2022 Feb 3;17(2):e0262993. doi: 10.1371/journal.pone.0262993 (PMC8812957; doi:10.1371/journal.pone.0262993)
Supplement: S1 Checklist — (DOCX) [file pone.0262993.s001.docx]

# Antimicrobial stewardship: Attitudes and practices of healthcare providers in selected health facilities in Uganda

**Supplementary information**

**STROBE Statement—Checklist of items that should be included in reports of *cross-sectional studies***

|  | Item No | Recommendation |
| --- | --- | --- |
| **Title and abstract** | 1 | (*a*) Indicate the study’s design with a commonly used term in the title or the abstract  **……………..................................... Page 2, line 6……………………………….....** |
|  |  | (*b*) Provide in the abstract an informative and balanced summary of what was done and what was found  **…………………………………. Page 2, lines 2-22………………………………..** |
| Introduction | | |
| Background/rationale | 2 | Explain the scientific background and rationale for the investigation being reported  **…………………………….. .pages 3-5, lines 26-87………………………………** |
| Objectives | 3 | State specific objectives, including any prespecified hypotheses  **………………... pages 2, lines 4-5………………pages 5……lines ….84-87** |
| Methods | | |
| Study design | 4 | Present key elements of study design early in the paper  **……………………………… page 6, lines 91-92 …………………………………** |
| Setting | 5 | Describe the setting, locations, and relevant dates, including periods of recruitment, exposure, follow-up, and data collection  **……………… page 6, lines 91-112… pages 10, lines 199-214……………………** |
| Participants | 6 | (*a*) Give the eligibility criteria, and the sources and methods of selection of participants  **………………………….. page 7, lines 115-123………………………………….** |
| Variables | 7 | Clearly define all outcomes, exposures, predictors, potential confounders, and effect modifiers. Give diagnostic criteria, if applicable  **…..page 10.….lines 182-197……………………………………..** |
| Data sources/ measurement | 8* | For each variable of interest, give sources of data and details of methods of assessment (measurement). Describe comparability of assessment methods if there is more than one group  **………………… page 10, lines 182-197………………………………………** |
| Bias | 9 | Describe any efforts to address potential sources of bias  **………………………. Page 12, lines 232-250……………………………….** |
| Study size | 10 | Explain how the study size was arrived at  **……………… page 7-8, lines 126-134……………..** |
| Quantitative variables | 11 | Explain how quantitative variables were handled in the analyses. If applicable, describe which groupings were chosen and why  **…………………… page 12, lines 230-233……………………….** |
| Statistical methods | 12 | (*a*) Describe all statistical methods, including those used to control for confounding  **………………..pages 12, lines 246-248………………………………..** |
|  |  | (*b*) Describe any methods used to examine subgroups and interactions  **…………………page 12, lines 146………………………………….** |
|  |  | (*c*) Explain how missing data were addressed  **…………………..page 12, lines 218-226………………………………..** |
|  |  | (*d*) If applicable, describe analytical methods taking account of sampling strategy  **………………….page 13, lines 249-250……………………………………** |
|  |  | (*e*) Describe any sensitivity analyses  **…………………………Not applicable………………………………………..** |
| Results | | |
| Participants | 13* | (a) Report numbers of individuals at each stage of study—eg numbers potentially eligible, examined for eligibility, confirmed eligible, included in the study, completing follow-up, and analysed  **……………Page 14, lines 264-269…………………………** |
|  |  | (b) Give reasons for non-participation at each stage  **………….page 6, line 124…**(Appendix 4 in S4 Appendix 4). **……………** |
|  |  | (c) Consider use of a flow diagram  **………** (S5 Appendix 5). **……….** |
| Descriptive data | 14* | (a) Give characteristics of study participants (eg demographic, clinical, social) and information on exposures and potential confounders  **…………………page 14, lines 264-272……………** |
|  |  | (b) Indicate number of participants with missing data for each variable of interest  **………………………186………………………………………………………..** |
| Outcome data | 15* | Report numbers of outcome events or summary measures  **………………………page 15, lines 275-280…………pages 21 line…312-317………………..** |
| Main results | 16 | (*a*) Give unadjusted estimates and, if applicable, confounder-adjusted estimates and their precision (eg, 95% confidence interval). Make clear which confounders were adjusted for and why they were included  **……pages 19 lines 293-307……………pages 23…, lines 327-337………….** |
|  |  | (*b*) Report category boundaries when continuous variables were categorized  **………………………………Page 8 ……lines 137-138…………………………** |
|  |  | (*c*) If relevant, consider translating estimates of relative risk into absolute risk for a meaningful time period  **………………………….we used odds ratios…………………………….** |
| Other analyses | 17 | Report other analyses done—eg analyses of subgroups and interactions, and sensitivity analyses  **…………………………… None …………………………………….** |
| Discussion | | |
| Key results | 18 | Summarise key results with reference to study objectives  **……..pages 25-28, lines 352-381, 383-421………………** |
| Limitations | 19 | Discuss limitations of the study, taking into account sources of potential bias or imprecision. Discuss both direction and magnitude of any potential bias  **……...............page29, lines 423-442………………………….** |
| Interpretation | 20 | Give a cautious overall interpretation of results considering objectives, limitations, multiplicity of analyses, results from similar studies, and other relevant evidence  **……………..pages 30, lines 445-451……………………..** |
| Generalisability | 21 | Discuss the generalisability (external validity) of the study results  **……………………page 29, lines 440-442………………………..** |
| Other information | | |
| Funding | 22 | Give the source of funding and the role of the funders for the present study and, if applicable, for the original study on which the present article is based  **……** **Makerere University-Swedish International Development Agency (SIDA) collaboration (Sida PI0010). The funders never participated in the study design, data collection and analysis, decision to publish, or manuscript preparation.** |

*Give information separately for exposed and unexposed groups.

**Note:** An Explanation and Elaboration article discusses each checklist item and gives methodological background and published examples of transparent reporting. The STROBE checklist is best used in conjunction with this article (freely available on the Web sites of PLoS Medicine at http://www.plosmedicine.org/, Annals of Internal Medicine at http://www.annals.org/, and Epidemiology at http://www.epidem.com/). Information on the STROBE Initiative is available at www.strobe-statement.org.
