# Supplementary figures and images for: Antimicrobial stewardship: Attitudes and practices of healthcare providers in selected health facilities in Uganda
Source: PLoS One. 2022 Feb 3;17(2):e0262993. doi: 10.1371/journal.pone.0262993 (PMC8812957; doi:10.1371/journal.pone.0262993)

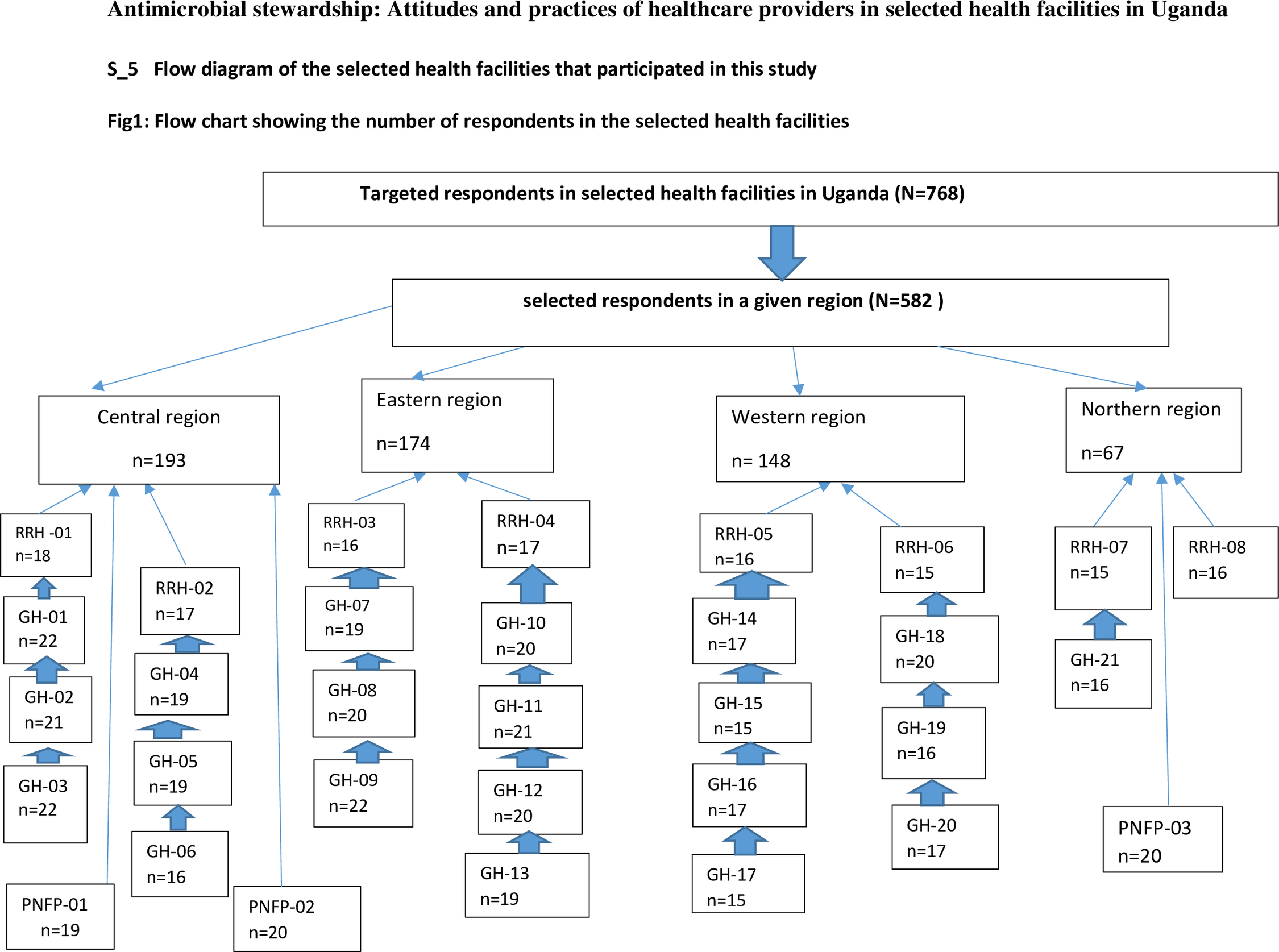

Supplement: S1 Fig — (TIF) [file pone.0262993.s002.tif]
