# Supplementary material for: Antimicrobial stewardship: Attitudes and practices of healthcare providers in selected health facilities in Uganda
Source: PLoS One. 2022 Feb 3;17(2):e0262993. doi: 10.1371/journal.pone.0262993 (PMC8812957; doi:10.1371/journal.pone.0262993)
Supplement: S2 Appendix — (DOCX) [file pone.0262993.s004.docx]

# Antimicrobial stewardship: Attitudes and practices of healthcare providers in selected health facilities in Uganda

**S4_appendix IV supporting information used in the tables of the manuscript**

Table 1 Sociodemographic characteristics of respondents (N=582)

|  | Frequency | Percentage (%) |
| --- | --- | --- |
| **Sex** |  |  |
| Male | 333 | 57.2 |
| Female | 249 | 42.8 |
| **Age categories** |  |  |
| 30-39 | 246 | 42.3 |
| 20-29 | 96 | 16.5 |
| 40-49 | 171 | 29.4 |
| 50+ | 69 | 11.9 |
| **Hospital department** |  |  |
| Medicine | 98 | 16.8 |
| Surgery | 71 | 12.2 |
| Paediatrics | 109 | 18.7 |
| Pharmacy | 50 | 8.6 |
| Obstetrics and Gynaecology | 93 | 16 |
| Outpatient Department | 101 | 17.4 |
| Other department | 60 | 10.3 |
| **Type of professional cadre** |  |  |
| Nurse | 199 | 34.2 |
| Pharmacy Technician | 30 | 5.2 |
| Clinical Officer | 136 | 23.4 |
| Medical doctor | 121 | 20.8 |
| Pharmacist | 24 | 4.1 |
| Medical specialist | 50 | 8.6 |
| Lab Technician | 22 | 3.8 |
| Total | 582 | 100 |
|  |  |  |
| **Level of academic training)** |  |  |
| Diploma | 327 | 56.2 |
| Degree | 191 | 32.8 |
| Masters and above | 64 | 11 |
| **years_at_work** |  |  |
| Less than 5 years | 184 | 31.6 |
| 5<10 | 140 | 24.1 |
| 10 and above | 258 | 44.3 |
| **Region of Uganda** |  |  |
| Central | 193 | 33.2 |
| North | 67 | 11.5 |
| East | 174 | 29.9 |
| West | 148 | 25.4 |
| **Type of health facility** |  |  |
| Regional referral hospital | 130 | 22.3 |
| General hospital | 396 | 68 |
| Private-not-for profit | 56 | 9.6 |
| **Nature of the health facility** |  |  |
| Teaching hospital | 186 | 32 |
| Non-teaching hospital | 396 | 68 |
| **Bed capacity** | N. | col% |
| 100 beds | 396 | 68 |
| 101- 300 | 37 | 6.4 |
| Over 300 | 149 | 25.6 |

**Table 2 showing the age distribution of study respondents**

| **Age (years)** | **N** | **sum** | **mean** | **sd** | **p50** | **p25** | **p75** |
| --- | --- | --- | --- | --- | --- | --- | --- |
|  | 582 | 22242 | 38.2 | 8.4 | 38 | 31 | 43 |

**Table 3 age distribution of study respondents by sex**

| **Age** |  |  |
| --- | --- | --- |
|  |  |  |
| **Male** | mean | 37.0 |
|  | standard deviation | 8.3 |
|  | median | 36 |
|  | Lower quartile | 30 |
|  | Upper quartile | 42 |
|  | minimum | 23 |
|  | Maximum | 60 |
|  |  |  |
| **Female** | mean | 39.8 |
|  | standard deviation | 8.2 |
|  | median | 39 |
|  | Lower quartile | 33 |
|  | Upper quartile | 43 |
|  | minimum | 20 |
|  | Maximum | 60 |
|  |  |  |
| **Total** | mean | 38.2 |
|  | standard deviation | 8.4 |
|  | median | 38 |
|  | Lower quartile | 31 |
|  | Upper quartile | 43 |
|  | minimum | 20 |
|  | Maximum | 60 |

**Table 4 Antimicrobial stewardship attitude score of healthcare providers (N=582)**

| **Healthcare professional Cadre** | **N** | **Sum of points scored** | **mean** | **sd** | **p50** | **p25** | **p75** |
| --- | --- | --- | --- | --- | --- | --- | --- |
| Nurse | 199 | 8851 | 44.5 | 11.4 | 48 | 42 | 52 |
| Pharmacy Technic | 30 | 1433 | 47.8 | 9.2 | 50 | 43 | 55 |
| Clinical Officer | 136 | 6160 | 45.3 | 11.0 | 48 | 42 | 53 |
| Medical doctor | 121 | 5717 | 47.2 | 10.9 | 51 | 45 | 54 |
| Pharmacist | 24 | 1264 | 52.7 | 4.0 | 53 | 50.5 | 55.5 |
| Medical speciality | 50 | 2375 | 47.5 | 11.0 | 50 | 45 | 55 |
| Lab Technician | 22 | 966 | 43.9 | 12.6 | 45.5 | 38 | 53 |
| **Total** | **582** | **26766** | **46.0** | **11.0** | **49** | **43** | **53** |

**Table 5 Antimicrobial stewardship practices scores of healthcare providers in Uganda**

| **Healthcare professional cadre** | **N** | **Sum of scored points** | **mean** | **sd** | **p50** | **p25** | **p75** |
| --- | --- | --- | --- | --- | --- | --- | --- |
| Nurse | 199 | 1514 | 7.6 | 3.0 | 8 | 6 | 10 |
| Pharmacy Technic | 30 | 213 | 7.1 | 2.8 | 8 | 5 | 9 |
| Clinical Officer | 136 | 1070 | 7.9 | 2.9 | 9 | 6 | 10 |
| Medical doctor | 121 | 967 | 8.0 | 2.6 | 9 | 6 | 10 |
| Pharmacist | 24 | 158 | 6.6 | 3.0 | 7 | 4.5 | 9 |
| Medical speciali | 50 | 356 | 7.1 | 2.7 | 8 | 5 | 9 |
| Lab Technician | 22 | 134 | 6.1 | 3.3 | 6 | 4 | 8 |
| **Total** | **582** | **4412** | **7.6** | **2.9** | **8** | **6** | **10** |

**Table 6. Blooms categorisation of AMS Attitude scores of the health care provider Professional cadre (N=582)**

| **5) Type of cadre** | **Poor** | **Average** | **Good** | **Total**  **N=582** |
| --- | --- | --- | --- | --- |
|  | **(n=52)** | **(n=190)** | **(n=340)** |  |
|  |  |  |  |  |
| Nurse | 23(11.5) | 72(36.2) | 104(52.3) | 199 |
| Pharmacy Technician | 1(3.3) | 11(36.7) | 18(60.0) | 30 |
| Clinical Officer | 12(8.8) | 49(36.0) | 75(55.2) | 136 |
| Medical doctor | 10(8.2) | 33(27.3) | 78(64.5) | 121 |
| Pharmacist | 0(0) | 2(8.3) | 22(91.7) | 24 |
| Medical specialist | 4(8.0) | 12(24.0) | 34(68.0) | 50 |
| Lab Technician | 2(9.1) | 11(50.0) | 9(40.9) | 22 |

**Table 7 Blooms categorisation of AMS Practices scores for healthcare providers professional cadre in health facilities in Uganda (N=582)**

|  | | | | |
| --- | --- | --- | --- | --- |
| 5) Type of cadre | Poor (n=143) | Average  (n=269) | Good  (n=170) | Total |
|  |  |  |  |  |
| Nurse | 47(23.6) | 91(45.7) | 61(30.7) | 199 |
| Pharmacy Technician | 8(26.7) | 17(56.7) | 5(16.6) | 30 |
| Clinical Officer | 30 (22.1) | 56(41.2) | 50(36.7) | 136 |
| Medical doctor | 23(19.0) | 62(51.2) | 36(29.8) | 121 |
| Pharmacist | 10(41.7) | 9(37.5) | 5(20.8) | 24 |
| Medical specialist | 15(30) | 26(52) | 9(18) | 50 |
| Lab Technician | 10(45) | 8(36) | 4(18) | 22 |
|  |  |  |  |  |

**Table 3. Predictors of antimicrobial stewardship (AMS) attitudes amongst healthcare providers in health facilities in Uganda (N=582) (**October 2019 to February 2020)**.**

|  | **Low score** | **Fair score** | **High scores** | **COR** | **AOR** | **P-value** |
| --- | --- | --- | --- | --- | --- | --- |
|  | **(n=52)** | **(n=190)** | **(n=340)** | **(95% CI)** | **(95% CI)** |  |
|  | **n(%)** | **n(%)** | **n(%)** |  |  |  |
| **Age (years)** |  |  |  |  |  |  |
| 20–29 | 13(25) | 29(15.3) | 54(15.9) | Reference group | Reference group |  |
| 30–39 | 16(30.8) | 77(40.5) | 153(45) | 1.48 (0.91- 2.39) | 1.46 (0.90–2.37) | 0.121 |
| 40–49 | 18(34.6) | 62(32.6) | 91(26.8) | 1.06 (0.64-1.77) | 1.05(0.63- 1.74) | 0.862 |
| 50+ | 5(9.6) | 22(11.6) | 42(12.4) | 1.59 (0.83- 3.02) | 1.57(0.83–2.99) | 0.168 |
| **Sex** |  |  |  |  |  |  |
| Male | 23(44.2) | 97(51.1) | 213(62.6) | Reference group | Reference group |  |
| Female | 29(55.8) | 93(48.9) | 127(37.4) | 0.65 (0.46–0.91) | 0.66 (0.47–0.92) | *0.016 |
| **Level of academic training** |  |  |  |  |  |  |
| Diploma | 36(69.2) | 124(65.3) | 167(49.1) | Reference group | Reference group |  |
| Degree | 13(25) | 51(26.8) | 127(37.4) | 1.77 (1.21–2.58) | 1.81 (1.24–2.63) | *0.002 |
| Masters and above | 3(5.8) | 15(7.9) | 46(13.5) | 1.96 (1.05–3.65) | 2.06 (1.13–3.75) | *0.018 |

**COR: Crude Odds Ratio, AOR: Adjusted Odds Ratio, CI: Confidence Interval**

*show significant difference at p < 0.05.

**Table 5. Predictors of antimicrobial stewardship practices amongst healthcare providers in health facilities in Uganda (N=582) (**October 2019 to February 2020**).**

|  | **Low score** | **Fair score** | **High scores** | **COR** | **AOR (95% CI)** | **P-value** |
| --- | --- | --- | --- | --- | --- | --- |
|  | **(n=133)** | **(n=261)** | **(n=166)** | **(95% CI)** |  |  |
|  | **n(%)** | **n(%)** | **n(%)** |  |  |  |
| **Age** |  |  |  |  |  |  |
| 30–39 | 20(21.7) | 46 (50) | 26(28.3) | Reference group | Reference group |  |
| 20–29 | 52(22.4) | 115(49.6) | 65(28) | 0.99 (0.62–1.6) | 1.02 (0.66–1.61) | 0.901 |
| 40–49 | 45(26.9) | 69(41.3) | 53(31.7) | 0.94 (0.64–1.38) | 0.95 (0.65–1.39) | 0.785 |
| 50+ | 16(23.3) | 31(44.9) | 22(31.9) | 1.11 (0.67–1.87) | 1.15 (0.69–1.91) | 0.593 |
| **Sex** |  |  |  |  |  |  |
| Male | 83(26.1) | 143(45) | 92(28.9) | Reference group | Reference group |  |
| Female | 50(20.7) | 118(48.8) | 74(30.6) | 1.13 (0.75–1.69) | 1.18(0.85–1.62) | 0.313 |
| **Region of Uganda** |  |  |  |  |  |  |
| Central | 37(20.2) | 91(49.7) | 55(30.1) | Reference group | Reference group |  |
| North | 10(15.6) | 29(45.3) | 25(39.1) | 1.52 (0.89–2.60) | 1.48(0.86–2.53) | 0.153 |
| East | 35(20.3) | 80(46.5) | 57(33.1) | 1.13(0.76–1.68) | 1.1 (0.75–1.64) | 0.615 |
| West | 51(36.2) | 61(43.3) | 29(20.6) | 0.54(0.35–0.82) | 0.52 (0.34–0.79) | *0.002 |

**COR: Crude Odds Ratio, AOR: Adjusted Odds Ratio, CI: Confidence Interval**

*shows a significant difference at p < 0.05.
