# Supplementary material for: Antimicrobial stewardship: Attitudes and practices of healthcare providers in selected health facilities in Uganda
Source: PLoS One. 2022 Feb 3;17(2):e0262993. doi: 10.1371/journal.pone.0262993 (PMC8812957; doi:10.1371/journal.pone.0262993)
Supplement: Attachment — Submitted filename: Response to reviewers .doc [file pone.0262993.s005.doc]

**RESPONSE TO REVIEWER'S COMMENTS ON MANUSCRIPT, PONE-D-21-10939**

Thanks for the valuable comments on our manuscript addressing these comments has helped improve our manuscript. Here below is a point-by-point response to the comments raised by the reviewers and the editor.

**Editor's** c**omment:** Please ensure that your manuscript meets PLOS ONE's style requirements, including those for file naming. The PLOS ONE style templates can be found at

<https://journals.plos.org/plosone/s/file?id=wjVg/PLOSOne_formatting_sample_main_body.pdf>

https://journals.plos.org/plosone/s/file?id=ba62/PLOSOne_formatting_sample_title_authors_affiliations.pdf

**Response to editor's comment #1:** Thanks for the comment. The format of the manuscript has been adjusted to suit PLoS ONE journal requirements as guided.

**Editor's comment #2:** Please include additional information regarding the survey or questionnaire used in the study and ensure that you have provided sufficient details that others could replicate the analyses. For instance, if you developed a questionnaire as part of this study and it is not under a copyright more restrictive than CC-BY, please include a copy, in both the original language and English, as Supporting information. Moreover, please include more details on how the questionnaire was pre-tested, and whether it was validated.

**Response to editor's comment #2:** Thanks for this comment. We have provided the questionnaire in supporting information

Lines 183-206, pages 10-11, we have provided additional information on the questionnaire development in the revised manuscript. Attached is a copy in the supplementary information, **https://osf.io/jqbzt/?view_only=d4c2aca2bedc40d6942a3a710ca2052b**

We performed an extensive literature review to identify existing studies that evaluated the attitude and practice of antimicrobial stewardship of healthcare providers in health facilities. Questionnaire items on AMS attitude included healthcare professionals' familiarity with AMS, the effectiveness of AMS in improving patient outcomes, patient safety, and reducing the spread of antibacterial resistance, all of which were adapted from previous studies on attitude. The questionnaire items on AMS practises included the following: adherence to standard treatment guidelines, culture and susceptibility testing, avoidance of excessive use of broad-spectrum antibacterials, and surgical antibacterial prophylaxis. These items were adapted from previous studies on AMS practices. The particular questions with items were not related to each other, so exploratory factor analysis was not applicable. The pharmacologist drafted the first version in English since it targeted formal healthcare providers who use English the questionnaire and invited experts in public health (1), epidemiology (1), microbiology (1), pharmacy (1), and pharmacology (1) to evaluate the instrument's face validity by looking at its clarity, ease of comprehension, arrangement or style, and effectiveness in capturing antimicrobial stewardship attitudes and practises. The same experts were invited to examine the questionnaire for readability, clarity, and comprehensiveness after reaching a consensus on which questions should be included in the preliminary final version of the questionnaire. A preliminary version was pilot-tested with 20 individuals from a convenience sample (doctors (6), nurses (4), allied health workers (8), pharmacists (2)). The respondents took about 30-45 minutes during the pilot to complete the questionnaire, and they provided feedback on any misleading or confusing question items. Participants suggested reformulating and rewording some items and deleting possibly repetitious ones. The final version of the questionnaire was developed using all submitted feedback from the pilot testing, where question items were arranged according to the respondents' breadth and depth of understanding and their respective hierarchies[36]. We further conducted reliability testing for a group of items on attitude and practice. The alpha Cronbach's coefficient was employed in the study to analyse the reliability and internal consistency of a set of questions about AMS attitude and practice. Cronbach alpha for AMS attitude was 0.9268, while for AMS practice, it was 0.762.

**Editor's comment #3:** We note that you have indicated that data from this study are available upon request. PLOS only allows data to be available upon request if there are legal or ethical restrictions on sharing data publicly. For information on unacceptable data access restrictions, please see [http://journals.plos.org/plosone/s/data-availability#loc-unacceptable-data-access-restrictions](http://journals.plos.org/plosone/s/data-availability#loc-unacceptable-data-access-restrictions).

**Response to editor's comment #3:** Data has been availed at the open science framework under this URL

DOI 10.17605/OSF.IO/ZX2RY

**https://osf.io/jqbzt/?view_only=d4c2aca2bedc40d6942a3a710ca2052b**

**Editor's comment #4:** If there are ethical or legal restrictions on sharing a de-identified data set, please explain them in detail (e.g., data contain potentially identifying or sensitive patient information) and who has imposed them (e.g., an ethics committee). Please also provide contact information for a data access committee, ethics committee, or other institutional body to which data requests may be sent.

**Response to editor's comment #4:** Thanks for the comment. There is no ethical or legal restriction on sharing a de-identified data set for our study. The de-identified data set for our study has been provided at the open science framework under URL, **DOI 10.17605/OSF.IO/ZX2RY**

**https://osf.io/jqbzt/?view_only=d4c2aca2bedc40d6942a3a710ca2052b**

**Editor's comment #5:** If there are no restrictions, please upload the minimal anonymised data set necessary to replicate your study findings as either Supporting Information files or to a stable, public repository and provide us with the relevant URLs, DOIs, or accession numbers. Please see <http://www.bmj.com/content/340/bmj.c181.long> for guidelines on how to de-identify and prepare clinical data for publication. For a list of acceptable repositories, please see [http://journals.plos.org/plosone/s/data-availability#loc-recommended-repositories](http://journals.plos.org/plosone/s/data-availability#loc-recommended-repositories).

**Response to editor's comment #5:** We have provided all supporting information on the open science framework under the following URL **https://osf.io/jqbzt/?view_only=d4c2aca2bedc40d6942a3a710ca2052b**

**DOI 10.17605/OSF.IO/ZX2RY**

**Editor's comment #6:** Your ethics statement should only appear in the Methods section of your manuscript. If your ethics statement is written in any section besides the Methods, please delete it from any other section.

**Response to editor's comment #6:** This has been deleted (Lines: 500, pages 31) and we have maintained the ethical statement only in the methods section of the revised manuscript (Lines: 297-306, pages 15).

**comment #7:** We note you have included a table to which you do not refer in the text of your manuscript. Please ensure that you refer to Table 5 in your text; if accepted, production will need this reference to link the reader to the Table.

**Response to editor's comment #7:** Thanks for the comment. Table 5 has been referred to in the revised manuscript. ( Lines 347, page 24)

**Editor's comment #8:** Please upload a copy of Supporting Information S5 which you refer to in your text on page 292.

**Response to editor's comment #8:** Thanks for the comment. We have uploaded supporting information S5 in the revised manuscript.

 We have uploaded supporting information for S5

https://osf.io/jqbzt/?view_only=d4c2aca2bedc40d6942a3a710ca2052b

**Comment Reviewer #1**: The authors present their findings on an important research topic – attitudes and practices around antimicrobial resistance in Uganda. This is an important study to help inform best practices for antimicrobial stewardship in Africa. However, I think that prior to publishing this work, additional modifications should be made to clarify the methods and results, which should be highlighted further in the discussion. I think the conclusions should also be reframed and toned down.

**Response:** Thanks for the comment, modifications on methods, results, discussion and conclusion in the revised manuscript.

**Comment Reviewer #1:** Study design: Cross-sectional, appropriate to the research question.

**Response:** Thanks for this valuable comment **Comment Reviewer #1:** Title: Appropriate, though it should be adjusted to note the study is about attitudes in children under 5 if that is the case.

**Response:** We modified the title of the questionnaire to match the subject being investigated. We have eliminated the part on children under five, which was deemed unrelated to this survey's subject matter (Check supplementary information on.
S3, https://osf.io/jqbzt/?view_only=d4c2aca2bedc40d6942a3a710ca2052b

**Comment Reviewer #1:** Abstract: Well-written and appropriate.

**Response:** Thanks for this valuable comment  **Comment Reviewer #1:** Introduction: Generally well-written and appropriate to the research material.

**Response:** Thanks for the comment

**Comment Reviewer #1:** Lines 41-44: Can you please specify what information is lacking? Have there ever been surveys of AMS attitudes and practices from sub-Saharan African countries? If so, what were the results? If not, please note this, and also note the other specific gaps in knowledge.

**Response: Lines 51-84:** We have improved the introduction with additional information from other studies in Africa. We have noted specific gaps in information from three studies from Zambia, Ethiopia and Nigeria. The adoption of AMS programmes has been slow and gradual among African countries, as evidenced by the literature, and several issues remain unaddressed. Only seven AMS programmes have been established in Africa. A top-to-bottom approach has been followed, disregarding the bottom-up approach that would have first assessed the attitudes and practices of healthcare providers on the ground before introducing AMS activities. In Zambia, Ethiopia and Nigeria, healthcare providers have demonstrated that they are familiar with AMS, wherein in both Ethiopia and Nigeria, healthcare providers demonstrated good AMS knowledge. However, when it came to assessing their attitudes, most had a casual and slack attitude towards antibacterial overuse as well as underrating the existence of AMR in their health facility, thus indicating a low attitude towards AMS. Most studies on attitudes and practices in Zambia, Nigeria, and Ethiopia have only focused on a few types of facilities, mostly one single type of health facility. They have excluded allied healthcare providers (clinical officers, pharmacy technicians, laboratory technicians), yet their practices and attitudes towards AMS may differ from one facility to another or within the facility itself. AMS attitudes may be affected by low staffing levels, inadequate government funding, and differences in infrastructure levels. The hierarchical nature of healthcare delivery may deny some healthcare facilities access to contextualised information on AMS, affecting their attitudes and practices. We implemented a study to assess AMS attitudes and practices among different healthcare cadres and indifferent health facilities.

**Comment Reviewer #1:** Line 53: Consider replacing the word 'nationwide' with another word – this study was not conducted at every hospital in Uganda.

**Response:** Thanks for the comment, we have removed the word 'nationwide' in the revised manuscript.

**Comment Reviewer #1:** Specify in the Methods or Results section what proportion of each type of hospital participated in the study.

**Response:** We have included the proportion of each different type of hospital that participated in the methods section (Lines 158-159 and 164-171, pages 9). We enrolled eight out of 16 (50%) regional referrals were enrolled, 21 out of 50 (42%) general hospitals and three out of four (75%) big private-not-for profit (PNFP ) health facilities.

**Comment Reviewer #1:** Lines 58-66: How was the recruitment/invitation to participate carried out? Was any compensation/incentive provided?

**Response:** Lines 229-233, pages 12: More information about the recruitment of participants has been added, as mentioned in the section below. Each facility had to first give us administrative approval before we could begin the study. The director's office provided us with a list of permanent workers as well as a breakdown of their duties.  Research assistants notified heads of departments and chairmen of other committees with letters about the study. The letters explained the purpose of the study, its objective, and who was eligible to participate in it. Recruitment phone calls were made to all respondents that were arranged in lists of the occupation groups after being availed with a list of employees provided by the medical director's office to conduct the recruitment process. Everyone was informed at the outset that their participation was entirely voluntary. After the respondent agreed to participate, the research assistant gave a brief explanation of the study's purpose, objectives, and procedures, and assured them that their participation was entirely voluntary, that they would remain anonymous, and that their information would be kept confidential. The information was gathered through the use of a questionnaire administered by an interviewer.

**Comment Reviewer #1:** Lines 59-62: Why were resident/MMed and other trainee physicians and midwives not included? In some hospitals, I imagine these are the people who may often prescribe or recommend antibiotics to patients.

**Response:** Lines 452-456: Thank you very much for bringing this to our attention. It is widely acknowledged that medical residents play an important role in the care of our patients and that they do prescribe a significant number of antibacterial drugs. However, because the majority of them work in teaching hospitals and are on a rotational basis, we classified them as part-time employees. Medical residents and medical interns were excluded from this study, which we believe is a limitation of the study. Midwives, general nurses, specialised nurses, and comprehensive nurses were all included in the definition of a nurse.

**Comment Reviewer #1:** Lines 69-71: Please describe the sample size method in more detail – referencing the WHO method does not make it clear how the appropriate number of hospitals and number of participants was calculated.

**Response:** Lines 147-159: The sample size description has been improved by including more detailed information on the formula used and the assumptions that were used to calculate it. We used a single population proportion formula to establish the sample size required for the healthcare providers participating in this study. There have been no previous studies on the prevalence of healthcare attitudes and practices in patients with AMS. As a result, we assumed that the proportion was 50% (P=0.5) to generate a significantly larger sample size. It has been projected that there are 42500 health care practitioners in the country. We obtained a sample size of 381 people by using a margin of error of 5%. The design effect was increased by 1.5 times to accommodate the sampling process, and a non-response rate of 11.5%, thus giving us a sample size of 768 respondents. We targeted a minimum of 24 health providers after dividing 768 by 32 health facilities. We modified the World Health Organisation (WHO method for assessing medication access, availability, and cost in health facilities to provide a framework for determining the number of health facilities in the region. We adopted the framework for selecting health facilities but didn't follow the recommended number or types of facilities. Thus, we increased the number of health facilities suggested to understand AMS attitudes better and practises in each area. We enrolled 32 health facilities, of which eight out of 16 (50%) were regional referral hospitals, 21 out of 50 (42%) general hospitals, and three out of four (75%) PNFPs in this study.

**Comment Reviewer #1:** Lines 73-74: Please add the proportion of the total facility type represented by the facilities sampled for this study (e.g. 8/16 RRH = 50%).

Response: Regional referrals, general hospitals, and PNFP proportions have all been added. We enlisted 32 health facilities in this study (Lines 156-158, Pages 8), including eight out of 16 (50%) regional referral hospitals, 21 out of 50 (42%) general hospitals, and three out of four (75%) PNFPs. We've also mentioned it on page 9,  lines 164, 168, and 169.

**Comment Reviewer #1:** Lines 89-90: How was the random sampling performed (e.g. what random selection algorithm was used, and how was it implemented)?

**Response:** Lines 162-180: We conducted our sampling using a list of hospitals compiled in a master list of health facilities maintained by the Ministry of Health. We created a regional referral sampling frame where each region was given its sampling pool of health facilities (five facilities in the Central, three in the Eastern, four in the Western, and four in the Northern). Using each region sampling frame, we selected two regional referrals for a region using a simple random sampling approach using the lottery method. Eight regional referral hospitals out of 16 50%) were chosen for the study. Using a broad hospital sampling frame from the Ministry of Health's master list of health facilities we enlisted all general hospitals in each region.

We selected 4 general hospitals for already selected regional referral health facilities using a two-criteria system: first, the selected general hospital had to refer patients to the regional referral, and second, it had to be within a one-hour ambulance drive of the same regional referral hospital. Using this two-criteria system, the study chose twenty-one general hospitals out of 50 (42%), where six were in the central, seven in the west, seven in the East, and one in the North). On the other hand, five general hospitals were eligible to participate, but we could not obtain administrative clearance due. Three out of four (75%) PNFPs graded at a regional referral health facility were sampled on purpose.

We targeted 24 healthcare providers in each of the 32 health facilities to achieve a sample size of 768 healthcare providers. We targeted 224 nurses, 192 clinical officers, 32 pharmacy technicians, 194 medical officers, 32 pharmacists, 64 medical experts, and 32 laboratory technicians out of the needed 768 healthcare providers. We needed one pharmacist, pharmacy technician and laboratory technician per participating health facility, while for the rest of the occupation groups (nurses, medical officers, clinical officers, medical specialists), we had to use the proportional number to size. We computed the proportionate number-to-size by dividing the number of professional cadres in a facility (x) over the research targeted professional cadres (y) multiplied by the minimal number in the health facility (z). We selected different numbers of healthcare providers per health facility (S5 supplementary information).

**Comment Reviewer #1:** Lines 93-95: Please be more specific about which questionnaire items were adopted from the literature, which have already been validated, and which were created de novo.

**Response:** Lines 183-205: these show more information on the adopted items of the questionnaires, how the face and content validation were conducted before piloting the questionnaire. We conducted in-depth evaluations of previous studies and reports on attitudes and behaviours related to antimicrobial stewardship. The questionnaire included; items on familiarity with AMS, the usefulness of AMS in improving patient outcomes, patient safety, and the prevention of antimicrobial resistance spread.

Additionally, we conduct reviews on previous studies on AMS to develop items relevant to healthcare provider behaviours about standard treatment guidelines, culture and susceptibility testing, avoidance of unnecessary broad-spectrum antibiotic use, and surgical antibacterial prophylaxis. We did not do exploratory analysis because the particular questions contained within the items (attitudes or practices) were unconnected.

We wrote the questionnaire entirely in English because it was aimed at healthcare providers that use English as their official language in their daily practice. The instrument's initial version was written by a pharmacologist. The instrument's face validity was evaluated by specialists in public health (1), epidemiology (1), microbiology (1), pharmacy (1), and pharmacology. They shared their perspectives on the clarity of each item's questions, the ease with which each item could be understood, and other suggestions for improving the interview's style and format. The same specialists were tasked with determining the instrument's content validity by assessing each item's completeness, readability, and comprehensiveness. The preliminary final version for the pilot study included sections on hospital characteristics, social demographics, and 12 items measuring AMS attitudes and practices. Before using it, we received expert consensus on each item of the questionnaire.

A preliminary version of the instrument was piloted in four hospitals with a convenience sample of twenty participants (doctors (6), nurses (4), allied health workers (8), and pharmacists (2). We used the tool to conduct an interview, which lasted approximately 30-45 minutes. The pilot participants offered comments on misleading or ambiguous question items. Certain item questions in the questionnaire were recommended to be restructured and reworded, while others were suggested to be deleted due to their potential for duplication.  The final version of the questionnaire was constructed by incorporating all of the feedback gained during pilot testing, and the experts further sorted question items sorted into hierarchies based on the breadth and depth of respondents' comprehension.  We were unable to retest the tool in the field after experts approved it was ready for use.

We assessed the reliability and internal consistency of a collection of questions regarding each AMS attitude and practice item using the alpha Cronbach's coefficient. The Cronbach's alpha was 0.9268 for AMS attitude and 0.762 for AMS practice.

**Comment Reviewer #1:** Line 110: Why did the authors choose a modified Bloom's categorisation? Has this been validated for this type of research question?

**Response:** We modified the Bloom classification to evaluate healthcare providers' attitudes and practises toward antimicrobial stewardship by grading healthcare providers' scores on a group of AMS attitudes and practises questions using scores of "high," "average," and "low" to classify healthcare providers in the questionnaire.

**Lines 213-216:** During the pilot testing, Bloom's categorisation allowed us to categorise the question items into breadth and depth based on our understanding of the respondent attitudes and practises on antimicrobial stewardship. It allowed us to structure the antimicrobial stewardship attitudes or practises questions items according to the hierarchy of understanding attitudes or practises. Finally, we graded healthcare practitioners' attitudes and practises toward antimicrobial stewardship in three categories (high, average, and low).

**Comment Reviewer #1:** Lines 111-113: I recommend reconsidering the labels of "good" and "bad" for the AMS score. These labels imply judgement about attitudes and practices. I recommend changing the labels to "high", "average", and "low" or something similar. Labeling people and their attitudes towards AMS as good and bad could have unintended negative consequences.

**Response:** Thanks for this valuable comment. These labels of "high", "average", and "low" have been adopted as recommended in the manuscript.

**Comment Reviewer #1:** Line 114: I don't think gender was assessed, only sex. Is this correct? If so, I recommend changing this to sex.

**Response:** Lines 234, Page 12: Gender has been corrected to sex in the revised manuscript.

**Comment Reviewer #1:** Lines 133-134: How were discrepancies in doubly-entered data resolved?

**Response:** Lines 245-250: The following information has been updated to provide greater clarification on how we dealt with discrepancies in data that had been entered twice. To undertake double data entry and validation, we used EpiDATA manager, which allowed us to enter survey questionnaires twice by two distinct data entrants at the same time. We later reviewed these files to see whether there were any conflicts or differences. Those variables that differed from one another were rigorously compared to the original tool from the field and harmonised as necessary to achieve the desired results. The procedure of validation was halted when the field tool and the input files appeared similar.

**Comment Reviewer #1:** Ethics lines 312-313: Why didn't all research participants give written informed consent to participate? I see a statement at the end of the manuscript that 'many' of the participants gave written informed consent, but not all.

**Response:**: We have deleted this section on page 32 and maintained the same section of ethics statements in the method (Lines 310-311). Every participant first consented before participating in the study

**Comment Reviewer #1:** I see the Appendix S3 notes the topic of the research is "Antimicrobial stewardship practices and quality of antibacterials use in children under-five in Health facilities of Uganda." Was this study specifically addressing antibacterial use in children? Or all patients?

**Response:** No, the manuscript addresses antimicrobial stewardship attitudes and practices in healthcare providers, while the entire doctoral study is about Antimicrobial stewardship practices and the quality of antibacterials use in children under five in Health facilities of Uganda.

Therefore, we have corrected the title of the questionnaire to harmonise with the title of the manuscript. (S3 supplementary information, <https://osf.io/jqbzt/?view_only=d4c2aca2bedc40d6942a3a710ca2052b>

**Comment Reviewer #1:** Lines 159-160: Were the non-respondents different to the respondents in any way (e.g. professional cadre, sex, age, etc)?

**Response:** They didn't differ at all since most non-responses emerged due to the failure of the hospital to give administrative clearance, which denied a similar number of healthcare providers to participate in the study.

**Comment Reviewer #1:** Line 171 and throughout the manuscript: Please see my note about labelling AMS scores as 'good' or 'bad'. The same is true for 'poor'. I think this should be changed to 'low'.

**Response:** As recommended by the reviewer, AMS score categories of high, average or low have been corrected throughout the manuscript.

**Comment Reviewer #1:** Lines 185-187: Please indicate directionality of the association, e.g., which level was positively or negatively associated with higher AMS score.

**Response:** Lines 316-322: We have improved our writing to communicate the association's direction better. Females (AOR: 0.66, 95% Cl: 0.47-0.92) were significantly less likely than males to have high AMS attitudes scores in the multivariate logistic regression model after controlling for confounding by including education and region in the model.  Furthermore, it was found that healthcare professionals with a degree (AOR: 1.81, 95% Cl: 1.24-2.63) were 1.8 times significantly more likely to have high AMS scores than those with diplomas. Healthcare professionals with a master (AOR: 2.06: 95% Cl: 1.24-2.63) were found to be 2.1 times more significantly likely than those with diplomas to score high AMS attitudes.

**Comment Reviewer #1:** Line 189: I recommend changing 'female' to 'male' and reversing the AOR, or changing the description of the association. What is written here implies that being female is associated with 'good' AMS attitudes, when in fact the AOR for female was 0.66, so it was 'protective' against a 'good' AMS attitude (which can be confusing to readers).

**Response:** Lines 316-318: We have changed the description to show the direction of the association. Females (AOR: 0.66, 95% Cl: 0.47-0.92) were found to be significantly less likely than males to have high AMS attitudes scores in the multivariate logistic regression model after controlling for confounding by including education and region in the model.

**Comment Reviewer #1:** Lines 212-213: Similar comment to my prior comment – the authors note that the Western region was a predictor of AMS practices, but the AOR is 0.52, indicating a negative association. Please clarify this in the text to indicate that it was significantly associated with a lower AMS practice score.

**Response:** Lines 345-348: Response: Lines 373-378: We have enhanced our writing to demonstrate the direction of the association's in our manuscript. Healthcare providers in the western region (AOR: 0.52, 95% CI: 0.34–0.79) had significantly lower AMS practise scores than those in the central region, according to the multivariable ordinal logistic regression results model.

**Comment Reviewer #1:** Lines 225-231: Are there any other differences between your study and the Ethiopian one that could explain this difference, e.g., years in which the study was carried out, study population, antibiotic availability, etc? Also, this Ethiopian study should probably be mentioned in the introduction as existing data.

**Response:** Lines 367 to 384: More information has been added to the manuscript to address this aspect

**Comment Reviewer #1:** Lines 232-233: Please see my note above about indicating the directionality of association. I think it would be clearer to note that male sex was associated with a higher AMS attitude score.

**Response:** Lines 390-397:The revised manuscript has been updated to reflect this change. According to the study's findings, the odds of having a high AMS attitude improved between 1.8 and 2.02 in our study when the healthcare professional held a bachelor's or masters' degree as opposed to those who held a diploma certification, according to the study results. Our findings showed that females had lower AMS attitude scores than males after adjusting for education and region. Since most respondents(56%) were females with the diploma-level academic level of training, possibly most of the diploma holders had not been exposed to AMS during their training and practice. The low AMS attitude scores of diploma holders and females could affect the implementation of AMS programmes in terms of comprehensiveness, quality, and adoption to lower community health facilities

**Comment Reviewer #1:** Lines 234-241: The way this association is described is troubling. Firstly, associating sex with good or bad attitudes can be a minefield – see my note above about changing this to high or low scores. Secondly, participant sex is likely confounded by professional cadre, making it difficult to draw inferences about this issue. However, the association of participant sex with the AMS attitude score was significant while adjusting for the level of education, which means that sex is associated with AMS attitude regardless of the level of education. Please examine this interpretation again closely and re-interpret it more carefully.

**Response:** Lines 390-397: The revised manuscript has been updated to reflect this change. Thank you for the comment. This study looked into whether there was a confounding effect between sex and education levels of healthcare providers on the outcome of the AMS attitude assessment. After assessing the association between education levels and the AMS attitude outcome, there was a statistically significant difference (P=0.002) between the two groups. We also observed a statistically significant difference (P=0.005) when we examined the association between sex and the outcome of the AMS attitude test. Our study looked at the association between sex and education and found a statistically significant difference (P 0.001). That means, education levels of healthcare providers confounds the relationship between sex and  AMS attitude outcome. Since we included education levels in the model, we could control for confounding variables in our model building.

**Comment Reviewer #1:** Additional discussion should be added about why Pharmacists and Pharmacy Techs have high AMS attitude scores but low AMS practice scores – it surprised me to see that their practices were much lower than average and much lower than the other professional cadres.

**Response:** We thank you for the observation, and we have added a note to the discussion.

Lines 450-460: we have added discussion on Pharmacists high AMS attitude and low AMS practices.

**Comment Reviewer #1:** Additional limitations should be added concerning the lack of data gathered from trainees and uncertainty around the cause and effect of AMS attitudes and practices.

**Response:** Lines 453-455: We have added additional information in the limitation section of the revised manuscript on the nature of study design, being unable to ascertain whether the high AMS attitude could have translated to AMS practices

**Comment Reviewer #1:** The ultimate conclusion – that more needs to be done to support AMS in Uganda – is a good one. However, I don't think it is justifiable to frame this conclusion in terms of cause and effect – that 'good' AMS attitudes are not causing 'good' AMS practices – as the authors know, this is association and not causation, and furthermore, there are many systemic barriers to implementing AMS practices regardless of the attitude. This should be acknowledged and the causal language adjusted or removed.

**Response:** Lines 461-462: Additional information has been added to the conclusion

In this study, most healthcare providers reported a high AMS attitude and average AMS practice. The Ministry of Health needs to do more work to support AMS activities at health facilities of Uganda.

**Comments Reviewer #2**:  Lines 33-50: Could authors add more information to motivate their study of AMS attitudes and practices of healthcare providers?

Is this the most important aspect of reducing antibacterial resistance in Uganda?

**Response:** Lines 30-84: There has been an increase in information on the function of AMS in optimising antibacterial use in several areas, particularly public health, where this study is based on the findings. Our discussion so far has focused on the critical role played by antimicrobial stewardship (AMS) programmes in optimising antibacterial use while still preventing the emergence of antimicrobial resistance.

We have also included the advocacy of the World Health Organization (WHO) and other international, national, and professional organisations to include antimicrobial stewardship (AMS) among the different pillars like infection control and medicine safety to strengthen health systems in low and middle-income nations. We have also shown the role played by AMS in ensuring optimal antibacterial use in other sectors like Agriculture.

Aside from that, we also discussed how slow progress was made in implementing the AMS programme and developing national action plans on antimicrobial resistance among African member countries following the endorsement of the World Health Organization's global action plan on antimicrobial resistance.

We have examined the impediment to the rapid adoption of AMS programmes that results from the top-to-bottom strategy, as opposed to the bottom-up approach, which incorporates a greater number of stakeholders. AMS programmes are not being implemented at community hospitals because of a lack of government support and contextualised information and the hierarchical nature of healthcare delivery, which excludes community hospitals from participating in the programmes. AMS attitudes and practices have been studied extensively in Zambia, Nigeria, and Ethiopia, but most of the research has had a limited scope in terms of geographical coverage, and most has excluded the Allied health workers who constitute the majority of the workforce at these health facilities.

A bottom-up approach was used to draft the National Antimicrobial Resistance Action Plan (2018-2023) and to form a national steering committee on antibacterial resistance. All relevant stakeholders from health facilities and other sectors were involved in this process, which was transparent and participatory. The execution of the National Antimicrobial Resistance Strategy (NAP) is in its latter stages, with the emphasis now on strengthening or implementing AMS programmes. The health ministry has formed an AMS National Steering Committee, which comprises stakeholders from health facilities and other sectors, to coordinate several initiatives, including enhancing or overseeing the implementation of AMS programmes at the health facility level. Although the Ministry of Health has maintained contact with heads of health institutions to strengthen existing medicine and therapeutic committees and antimicrobial stewardship programmes, the attitudes and practices of healthcare providers toward antimicrobial resistance (AMS) remain unknown in all four Ugandan regions. Therefore, the current study investigated healthcare providers' attitudes and practices regarding AMS and associated factors in Uganda's regional referral hospitals, general hospitals, and private-not-for-profit (PNFP).

.**Comments Reviewer #2**:  Is the paucity of information on this specific aspect? How was the National Action Plan on Antimicrobial Resistance drafted; bottom-up or top-to-bottom? When drafted? Is it implemented? If yes, how and when (thanks, some information is provided in the Discussion section (lines 227-229) but it needs to be clear from the start) …

**Response:** Lines 86-95: This information is provided by the National Action Plan on Antimicrobial Resistance (NAP on AMR) has been improved. The drafting of the national action plan on antimicrobial resistance took place using a bottom-up approach, with key participants selected from representative sectors and health facilities to contextualise the global action plan for antimicrobial resistance within Uganda's context. The later formation of the AMS national steering committee was attended by representatives from the infection control committee and the medicine and therapeutics committees, who enabled the contextualising of NAP in the ongoing AMS activities of the health facilities.

**Comments Reviewer #2**:  Lines 53-57: Could authors provide more information about the characteristics of these different hospitals, such as their bed size, teaching status, location characteristics (urban or rural…), their regional distribution etc? Are there any for-profit hospitals in Uganda?

**Response:** Line 101-133: Uganda has an integrated health system with public and private health facility services spread across 146 districts, 312 counties, 2184 sub-counties/towns/municipalities/divisions, 10595 parishes, and 70626 villages.

We concentrated our study on public hospitals, which account for 45% of the total, and PNFP, which accounts for 40%. These health facilities receive government support and are included in most health-system-strengthening initiatives. The study included regional referral and general hospitals among public health institutions, whereas the PNFP hierarchy included the big PNFP classed as regional referrals.

We included regional referral and general hospitals and the four PNFPs since they have benefited from ongoing government programmes, coordinated by the Ministry of Health and other development partners, to enhance the supply chain and medicine use at these health institutions. As part of a health system strengthening effort to optimise antibacterial use, several trainings have been given to enhance medicine and therapeutic committees, infection prevention and control, and antimicrobial stewardship. Because antibiotic use cuts across all fields and health facilities, we expanded the study's geographic scope to improve the generalizability of our findings. We chose health institutions in all of Uganda's regions to maximise the generalizability of the study's findings. In Uganda's four regions, the study covered regional referral, general hospitals, and PNFPs. The number of districts, population, and health facilities varied between the areas. We omitted national referral hospitals since they were undergoing renovations at the time of the study, and staff were dispersed across several referral health facilities.

Over nine million people live in the central region, which is divided into 24 districts. Mulago Hospital, with 1500 beds, and Butabika Hospital, with 550 beds, are the two national referral hospitals. Five regional referral hospitals (Masaka (330), Mubende (170), China-Uganda friendship hospital-Naguru (100), Kayunga (300), and Old Mulago) serve the region, receiving patients from eight general hospitals (Rakai, Nakaseke, Kalisizo, Kawolo, Kiboga, Lyantonde, Mityana, and Mpigi), each with 100 beds.

Uganda's Eastern region is divided into 30 districts, with a total population of 9,042,422. The regional referral hospitals in Mbale, Jinja, and Soroti have 400, 408, and 250 beds, respectively. These facilities provide specialist services to the region as well as thirteen conventional hospitals with a total capacity of 100 beds (Aturtur, Bududa, Bugiri, Bukwo, Busolwe, Iganga, Kamuli, Kapchorwa, Katakwi, Masafu, Pallisa, Buwenge, and Tororo).

There are 26 districts in the Western area, with a combined population of eight million people. Mbarara (600 beds), Fort Portal (333 beds), Kabale (280 beds), and Hoima (208 beds) are the four regional referral hospitals, each with 100 beds, that provide specialised care to patients referred from the nine general hospitals (Bundibugyo, Itojo, Kagadi, Kasese, Kiryandongo, Kyenjojo, Kilembe, Masindi, and Kitagata).

There are 29 districts in the northern region, with a population of seven million people. Four regional referral hospitals (Arua (272 beds), Lira (346 beds), Gulu (250 beds), and Moroto (114 beds) serve eleven general hospitals (Abim, Adjumani, Anaka, Kuluva, Kabong, Kalong, Kitgum, Moyo, Maracha, Nebbi, Yumbe). There are 44 PNFP hospitals in Uganda, although only four of them are designated as regional referral centres (St Mary's Hospital (Lacor), Lubaga, Mengo, and Nsambya).

**Comments Reviewer #2**:  Lines 53-57: Could authors explain their reasons for their focus on hospitals only? Are the AMS programs focus only on hospitals?

**Response:** We have added more information on why this study focused on public hospitals (regional referrals, general hospitals) and large PNFPs.

**Comments Reviewer #2**:  Lines 53-57: Please provide information about the characteristics of regions in Uganda.

**Response:** We have characterised the regions in terms of the distribution of the population, number of districts and the distribution of public hospitals (regional referral and general hospitals) and PNFPS.

**Comments Reviewer #2**:  Lines 69-71: Thanks for the information. This publication requires that at least 3 "sectors" be identified as a 1st step. So, as before, which are the important "sectors" for the subject matter of this study, only hospitals?

**Response:** Lines 41-44: We have provided this information on sectors served by AMS in the manuscript

**Comments Reviewer #2**:  Lines 71-74: Thanks for the information. However, it is unclear to the readership how the increase from 4 to 8 regional facilities should be interpreted? The recommendation of "4" is per sector in the publication. Again, are there other "sectors" that are of importance for the subject matter?

**Response:** Lines 153-157: We have increased clarity of the information to provide a rationale for increasing regional referral and general hospital to fit the context of Uganda's referral system.

**Comments Reviewer #2**:  Lines 74-79: Could authors provide reasons for targeting 24 healthcare providers in each of the hospitals? Are all of these hospitals the same size?

**Response:** Lines	172-180: Since 65% of studied health facilities were general hospitals with the same recruited number of healthcare providers. We were also aware that some workers resign or go for further studies, which may affect the constant number. Additionally, in terms of establishment and type of health facility, regional referrals and PNFPs have more staff from different occupation groups than general hospitals. We decided to use the national ratios of public healthcare workers to derive the targeted respondents in occupation groups. After generating the targeted numbers of respondents, we used proportional numbers-to-size to create numbers in each occupational group to interview in each facility.

We targeted 24 healthcare providers in each of the 32 health facilities to achieve a sample size of 768 healthcare providers. We targeted 224 nurses, 192 clinical officers, 32 pharmacy technicians, 194 medical officers, 32 pharmacists, 64 medical experts, and 32 laboratory technicians out of the needed 768 healthcare providers. We needed one pharmacist, pharmacy technician and laboratory technician per participating health facility, while for the rest of the occupation groups (nurses, medical officers, clinical officers, medical specialists), we had to use the proportional number to size. We computed the proportionate number-to-size by dividing the number of professional cadres in a facility (x) over the research targeted professional cadres (y) multiplied by the minimal number in the health facility (z). We selected different numbers of healthcare providers per health facility (S5 supplementary information).

**Comments Reviewer #2**:  Line 82: Again, what are the characteristics of these 4 regions?

**Response:** Lines 102-137: we have added information on the characteristics of the four regions in terms of populations, number of districts, number of regional referrals, general hospitals and PNFPs.

**Comments Reviewer #2**:  Lines 82-86: Please be clear if this means that none of the 3 "national referral hospitals" (lines 54-55) were selected into the sample. If so, please provide the rationale.

**Response:** Lines 105-107: We didn't include national referrals since there were undergoing renovation and many structural and human resource restructuring were ongoing at the time of the study. On the other hand, most of the strengthening of medicines and therapeutic committees that promote AMS activities focused on public health facilities (regional referral, general hospitals) and the large four private-not-for-profit not-for-profit referrals graded with a regional referral. We chose three groups to measure the impact of the ongoing activities.

**Comments Reviewer #2**:  Lines 82-83: Again, how are the 16 regional referral hospitals (line 56) distributed in these 4 regions?

**Response:** Lines 110-133: We have added more information in the manuscript on the distribution of regional referral health facilities in the four regions

The central region is served by five regional referral hospitals (Masaka (330beds ), Mubende (170beds), China-Uganda friendship hospital-Naguru (100beds), Kayunga (300beds) and Old Mulago (1000),

Three regional referral hospitals serve the eastern region; Mbale, Jinja, and Soroti, with 400, 408, and 250 beds.

The western region has four regional referral hospitals: Mbarara (600 beds), Fort Portal (333 beds), Kabale (280 beds), and Hoima (208 beds), which provide specialised care.

The northern region has 29 districts with a population of seven million people. There are four regional referral hospitals (Arua (272 beds), Lira (346), Gulu (250), and Moroto(114))

**Comments Reviewer #2**:  Lines 83-85: How are the "50 general (district) hospitals" (line 56) distributed in these 4 regions?

**Response:** Lines 102-139: We have added information on the distribution of general hospitals in the four regions.

The Eastern region has 14 general hospitals with 100 beds (Aturtur, Bududa, Bugiri, Bukwo, Busolwe, Iganga, Kamuli, Mbale regional cancer centre, Kapchorwa, Katakwi, Masafu, Pallisa, Buwenge, and Tororo).

The Central regions are served by which receive patients from 11 general hospitals (Entebbe, Kiruddu, Kawempe, Rakai, Nakaseke, Kalisizo, Kawolo, Kiboga, Lyantonde, Mityana, Mpigi) with 100 beds.

The Western region is served by 11 general hospitals (Bundibugyo, Itojo, Bwera, Kagadi, Kasese, Kiryandongo, Mbarara regional cancer centre, Kyenjojo, Kilembe, Masindi and Kitagata) with 100 beds.

The northern region has 13 general hospitals with 100 beds (Abim, Arua regional cancer centre, Maracha, Gulu regional cancer centre, Kuluva, Adjumani, Anaka, Kabong, Kitgum, Moyo, Maracha, Nebbi, Yumbe).

**Comments Reviewer #2**:  Lines 83-85: Please be clear how 3 "general hospitals" in each to the 4 regions add up to "21 general hospitals" (lines 72-73 or line 300) and, therefore, to "32 health facilities" overall (lines 73-74 or Abstract, line 15) for the study?

**Response:** Lines 165-170: We have provided more additional information on the selection of the general hospitals using a two criteria system.

**Comments Reviewer #2**:  Lines 85-86: Could authors provide information about the regional distribution and characteristics of the 4 "private-not-for-profit health facilities"?

**Response:** Lines 133-134: We have provided this information on distributing the private-not-for-profit graded as regional referrals. There are 44 PNFP hospitals in Uganda, although only four are graded as regional referral centres (St Mary's hospital (Lacor), Lubaga, Mengo, and Nsambya). Three are located in the Central (Nsambya, Lubaga, Mengo) and one in the North (Lacor).

**Comments Reviewer #2**:  Lines 87-88: Please reconcile "departmental heads" here with 4 "heads of departments" above (lines 77-78).

**Response:** Lines 197-198: We have corrected this information in the manuscript.

**Comments Reviewer #2**:  Lines 88-90: Again, do all the facilities have the same number of healthcare providers?

**Response:** Lines 176-182: We used 24 as an arbitrary total number of healthcare providers we needed from the 32 health facilities after generating our targeted sample size of 768. We targeted in all 32 health facilities over 224  nurses, 192 clinical officers, 32 pharmacy technicians, 32 pharmacists, 192 medical officers, 64 medical specialists, and 32 laboratory technicians. We needed one pharmacist, pharmacy technician and laboratory technician per participating health facility, while for the rest of the occupation groups (nurses, medical officers, clinical officers, medical specialists), we had to use the proportional number to size. We computed the proportionate number-to-size by dividing the number of professional cadres in a facility (x) over the research targeted professional cadres (y) multiplied by the minimal number in the health facility (z). We selected different numbers of healthcare providers per health facility (S5 supplementary information).

**Comments Reviewer #2**:  Lines 130-135: Please be clear if this means that there were no missing data items. If there were missing data items, what procedures were followed?

**Response:** Lines 243-246: we improved the manuscript on how missing data were handled from the health facility up to data analysis. During data collection in each hospital, the supervisor reviewed every case report form to ensure that every variable was filled. If more than four variables were missing data, the research assistant was requested to contact the respondent for more data. We performed a complete case analysis during data cleaning and deleted cases that had important missing variable data. In case of missing data items on a respondent's questionnaire, the research assistant ensured they contacted the healthcare provider to complete the questionnaire.

**Comments Reviewer #2**:  Lines 159-160: Again, please address "768" potential respondents in light of the number of hospitals included in the study sample. As above, what was the number of hospitals in the sample; "32" or 23?

**Response: 159-160:**We have added more information on the number of hospitals in the study
**Comments Reviewer #2**:  Lines 159-218: The reviewer finds it impossible to interpret the results without clarifications for the points above.

**Response:** Lines 159-160: we added 32 health facilities to indicate where we enrolled our study respondents.

**Comments Reviewer #2**:  Lines 227-229: Again, could authors make clear from the very beginning what was the rationale for their study, in general, and the survey, especially the "AMS practice" part, if "the Ministry of Health in Uganda has not implemented any formal interventions on AMS among healthcare providers"?

**Response:** Lines 360-367: More clarification on the study rationale has been provided in the manuscript

**Comments Reviewer #2**:  Please avoid typos and ensure completeness and transparency in the manuscript: a) please ensure that all of the acronyms (such as, AMS, in line 25) are spelled out the first time they are mentioned (it is in the Abstract but also needs to be spelled out in the body of the manuscript), b) please ensure that the references are complete (such as #18, for example, please be clear about "WHO" and the location of the publisher…), c) "would; reduce" (?) in line 173 or "is has been" (?) in line 257, d) please ensure that tables are self-explanatory (see, for example, Table 2, what are "AMS" and "60 points" & what does the second note refer to, or Table 4, why "HCP"…)

**Response:** Thanks for the advice, We have improved tables to be self-explanatory

**Comment Reviewer #3:** Please do not start the sentence with an acronym. Also, specify what AMS stands for.

**Response:** Line 33-34: The acronym has been written in full.

**Comment Reviewer #3:** I would suggest organising the introduction better. I think that it is a little confusing, too many acronyms, and the concepts are mixed. It would be appropriate to outline the context the research refers to and then mention what happened in other countries.

**Response:** Thanks for the comment. The context of antimicrobial stewardship attitudes and practices in other sub-Saharan countries has been rewritten.

**Comment Reviewer #3:** Subsequently, I would analyse the problem spread of the antibacterial resistance and the importance of AMS programmes in Uganda.

**Response:** Lines 33-84: We have improved the writing to show the importance of AMS in optimising antibacterial use and controlling the spread of antibacterial resistance.

**Comment Reviewer #3:** Why did the authors exclude those who had worked for only one year in the health facility?

What was the rationale?

**Response:** During the pilot of the study, most respondents who had been appointed as staff within one year would not complete the questionnaire due to inadequate information on AMS activities in the hospitals

**Comment Reviewer #3:** Authors used ordinal logistic regression to model the data (outcomes were divided into 3 categories). Was the proportional odds assumption checked?

**Response:** Lines 290-296: We have indicated how we checked proportional odds assumption of two independent variables (nature of teaching health facility and bed capacity) which indicated multicollinearity between these two variables.

**Comment Reviewer #3:** How were variables selected into the models? How was model fit assessed?

**Response:** Lines 263-273: we show how the backward selection method alongside the logical approach, where all variables with a p-value less than 0.2 at bivariable analysis were considered for inclusion in the multivariable model. Sex and age were considered universal confounders; even if they did not meet the 0.2 significance threshold, they were included. The dependent variable had three categories; "low," "average" and "high" attitude or practice scores of AMS.

**Comment Reviewer #3:** In the discussion section it seems redundant to define what was done in this study and repeat the results.

**Response:** Lines 367-445: We have improved the discussion section by adding the implication of our findings.

**Comment Reviewer #3:** It is correct instead of the comparison with other findings, as the authors did, even though this section should be expanded to present a broader context in which this research is relevant (there are only a few citations to Ethiopia).

**Response:** Lines 390-413: We have improved this section to elaborate why our repondents in our study had a high AMS attitude.

**Comment Reviewer #3:** For instance, results should be interpreted within the perspective of antimicrobial resistance and healthcare-associated infections, considering both low-middle income countries and around the world. Relevant articles worth including may be PMIDs 34223045, 32062724, 33961678, 33882843, 29590400, and 34213520.

**Response:** We have added these relevant articles. We are so grateful.

Thanks for sharing this information. Have gladly reviewed and added it to the manuscript
