# Supplementary material for: Antimicrobial stewardship: Attitudes and practices of healthcare providers in selected health facilities in Uganda
Source: PLoS One. 2022 Feb 3;17(2):e0262993. doi: 10.1371/journal.pone.0262993 (PMC8812957; doi:10.1371/journal.pone.0262993)
Supplement: Attachment — Submitted filename: Response to reviewers .docx [file pone.0262993.s006.docx]

**RESPONSE TO REVIEWER'S COMMENTS ON MANUSCRIPT, PONE-D-21-10939R1**

Dear Editor and reviewers:

**We greatly appreciate the thorough and thoughtful comments provided on our submitted manuscript. We have revised our manuscript according to the reviewers' comments, questions, and suggestions. Please see below our detailed responses to the reviewers' comments. All our responses are in bold. Please let us know if you still have any questions or concerns about the manuscript, and we will be happy to address them promptly.**

**Yours,**

**The authors of PONE-D-21-10939R1**

**Editor's** c**omment:**

Thank you to the authors for their work on revising the manuscript so far. Could the authors now focus on the point below, explaining what choices they made when administering the surveys and clarifying the rationale behind such choices?

**Response to Editor's comment #1:**

**We choose different types of health facilities for this study because of a gap in the literature on attitudes and practices of healthcare providers between urban regional referral hospitals, private not for profit (PNFP) hospitals and community general hospitals. Previous studies in Nigeria and Ethiopia had concentrated on studying only tertiary or specialised hospitals, limiting their generalizability. The study hospitals were selected due to the presence of structures and systems for strengthening the monitoring and improvement of medicine use. Most government and development partners capacity building has focused on establishing infection prevention committees, medicines and therapeutic committees in these hospitals.**

**We chose a large sample size of healthcare providers to increase the study's representativeness and determine the predictors of AMS attitude and practices in health facilities in Uganda.**

**We chose the administration office and heads of department to recruit the healthcare providers into our study. We used emails, letters, and phone calls to recruit healthcare providers to increase the participation of healthcare providers.**

**Every participant consented first before being interviewed.**

**We used an interview-administered questionnaire. We used research assistants from the same facility to administer the questionnaire to minimise bias in the responses. During piloting, we realised respondents gave different responses when the principal investigator or research assistant interviewed, so we used a research assistant other than the investigator himself to minimise the measurement bias. We used research assistants from different health backgrounds within the health facility to avoid the interviewer social desirability effect.**

**Editor's** c**omment:**

The manuscripts provides a good amount of information about, for example, the healthcare worker population, hospitals, and regions. It does not appear as though this info has been used to devise an appropriate cluster sampling, which would have in turn guided the analysis of data.

**Response to Editor's comment #2:**

**However, much as we couldn't conduct cluster sampling, the study catered for clustering at the healthcare provider level at the health facility**

**Editor's** c**omment:**

Although the authors calculated an overall sample size, this was not used to administer the surveys randomly around the country, as the administration ultimately does not appear random, but rather distributed to certain (types of) hospitals in some regions.

**Response to Editor's comment #3:**

**Yes, we agree with the Editor's comments. We could not use random sampling to all types of hospitals since we had to receive administrative clearance to know the sampling frame of participating hospitals. We randomly selected eight out of 16 regional referrals, where two regional referrals were picked per region to increase regional representativeness. All eight out 16 had granted us administrative clearance. However, of all the 32 out of 47 general hospitals selected after random sampling, only 21 granted us administrative clearance to conduct the study. Eleven general hospitals didn't participate in the selected sample.**

**We could not randomly select pharmacists, pharmacy technicians, and laboratory technicians in all hospitals that participated since each hospital had only one employee of these types of professional cadre. However, nurses, medical officers, clinical officers and medical specialists were selected randomly.**

**Reviewer #1**

This revised version is significantly improved from prior. I recommend some additional minor changes prior to publication, which I have noted below.

**Response:**

**Thanks for the valuable comments, and we appreciate your time.**

Methods and Results:
Largely re-written. There is excessive detail on the number of hospitals in each region which is somewhat distracting to the reader.

**Response:**

**Lines 99-107 on page 6; We have improved the clarity of writing in this section, deleted names of hospitals and limited ourselves to the number of targeted hospitals and the reasons for their selection.**

The authors still have not described how recruitment/invitation to participate was carried out at each facility to reach the enrollment targets and whether compensation/incentive was provided.

**Response:**

**Lines 197-205, page 11. We apologise for that, and we have added more information on how the research assistant conducted recruitment. We have also added more information on compensation for time and transport since some respondents were on leave and were requested to come to the facility**

In my prior comments, I suggested categorising the AMS attitude scores as 'high', 'average', and 'low'. Re-reading the manuscript now, I think 'average' is not the right label, as this has statistical meaning as well. Consider using 'moderate' or 'fair' to label this category instead of 'average'.

**Response:**

**The authors want to thank the review and have agreed to use "fair" instead of "average", as reflected in this manuscript's sections.**

**Comment Reviewer #1**:

Results of statistical models have been re-written and are more appropriate. In Line 348, I recommend changing the start of the sentence to 'In multivariable models adjusting for possible confounders, AMS practice scores …' This is to clarify that you are referring to your model results. Also include the AOR and its associated confidence interval in this sentence, not just the P-value for the association.

**Response:**

**Lines 321-323, page 23-24. We have corrected it as suggested.**

**Comment Reviewer #1**: 
Discussion and Conclusions:
Largely re-written. In line 364 I recommend avoiding the phrase 'will never', because this is unlikely to be the case.

**Response:**

**Lines 333-334, pages 25, Thanks for the correction. We have improved the clarity of the statement**

Consider rephrasing as 'are unlikely to'. In addition, there is only one limitation addressed – about potential bias in the interview. I think additional limitations should be added concerning the lack of data gathered from trainees (though part-time employees, they do prescribe a significant proportion of antibiotics and have their own AMS attitudes and practices, which were not measured here), uncertainty around the cause and effect of AMS attitudes and practices, and potential lack of generalizability of your results to non-Ugandan or non-East African settings.

**Response:**

**Lines 417-419, page 29. We have included the limitation of part and intern healthcare providers.**

**Lines 422-423, Pages 29. We have added a limitation on the uncertainty surrounding the cause and effect of AMS attitudes and practices.**

**Lines 423-424, Pages 29: We have added an aspect on our results not being generalisable to other non-East African settings or non-Ugandan setting**

**Reviewer #2**

Reviewer #2: Thanks for an improved manuscript. The following needs to be taken into consideration.
**Response:**

**Thanks for the valuable comments; we appreciate**

**Comment Reviewer #2**: 
Thanks for revisions; the Introduction section motivates the study well. However, it is now more than three pages long. Please consider shortening it without losing any of its content.

**Response:**

**Lines 26-92, We have shortened the introduction section and its now two pages.**

**Comment Reviewer #2**: 

Thanks for information about 2 (or is it 3?) "national referral" hospitals (new lines 102-105) that were excluded. a)As a result, (and names are not required but) what % of the public health hospitals were excluded?

**Response:**

**Lines 86-107, pages 6. We have clarified the health system characteristics. In our clarification, we report that the health system comprises 6937 health facilities, and of these, 3133 (45.6%) are public health facilities. The composition of the latter includes two national referral hospitals, three referral hospitals (the Uganda Cancer Institute, the Uganda Heart Institute, and the Women's Hospital), 13 regional referral hospitals, 47 general hospitals, 166 level IV health centres, 962 level III health centres, and 1321 level II health centres. We had only 63 out of 3133 (2%) health facilities participating. We excluded 3070 health facilities. The percentage of health facilities excluded is 98% (3070/3133).**

(a) "two million" (line 107)?

**Response:**

**Lines 86-107, pages 6. Thanks for this highlight. According to the health facilities of Uganda Master list (2018) page 7, Each health facility has a designated population size that it is meant to serve; a national referral hospital (10,000,000 persons), each regional referral hospital (2,000,000 persons), general hospitals (500,000) and, Health centre IV (100,000), Health centre III (20000), health centre II (5000) and health centre I (not defined).**

**In our previous description of the study setting, we had included this characteristic showing that a regional referral serves over two million persons; however, we have removed it in the revised manuscript for clarity.**

(c) Which region is this; (again, we do not need names and the detail but) why "five regional referral hospitals" followed with 4 names (lines 108-109)?

**Response:**

**Lines 99-107, pages 6. We thank you for this observation and the need for clarification. In the central region, we have five regional referrals hospitals that we included in our study.**

**Comment Reviewer #2**: 
(d) What does "approximately 50 general hospitals" (line 116) mean or are there 41 (lines 115-124) general hospitals? (e) Please be clear, do these hospitals have about 10 beds each (lines 123-124) or 100 beds? (f) If there are "44" private not-for-profit (PNFP) hospitals and if "3 out of 4" that are considered as "regional referral centres" were included in this study, what % of PNFPs was excluded from this study?

**Response:**

**Lines 95-107, page 6. In the PNFP health systems, there are 1009 health facilities with the highest referral level in the four tertiary hospitals, followed by 40 general hospitals and 955 health centres. We excluded all the 40 general PNFP hospitals and 955 health centres making a percentage of 98.6%.**

**Comment Reviewer #2**: 
3. "Old Lines 53-57: Could authors explain their reasons for their focus on hospitals only? Are the AMS programmes focus at hospitals?"
Thanks for explanations. In the healthcare system, are hospitals the only healthcare institution type where antibacterial misuse is observed?

**Response:**

**Lines 102-108, page 6. Thanks for the comment. We agree with you that overuse and misuse of antibacterials are not limited to regional referrals, general hospitals and PNFPs. Recent health initiatives have focused on training most public health facilities, tertiary PNFP healthcare providers, and private healthcare providers in communities on antimicrobial resistance and antimicrobial stewardship. Indeed most healthcare providers have been trained on the implementation of antimicrobial stewardship interventions. There has been the ongoing implementation of the National action plan, and the focus of the government has been on strengthening existing or initiating medicines and therapeutic committees. The committees implement antimicrobial stewardship, pharmacovigilance, and supply chain management in regional referrals, general hospitals, and PNFPs. Our study focused on assessing the attitudes and practices of healthcare providers on the implementation of these programs. However, further initiatives are needed to understand the attitudes and practices of healthcare workers in the lower health facilities since there are many challenges like infrastructure, human resources and follow of information.**

**Comment Reviewer #2**: 
This section in its new format now indicates that information about total number of observational units were available to authors. Was this information also available at the hospital level?

**Response:**

**Lines 130-141, page 8. Thanks for this comment. We could not know how many hospitals we shall collect from data not until the hospitals granted ethical and administrative clearance. We did not have information at the hospital level because the ethical guidelines in Uganda required the researcher to be issued ethical clearance from the parent institution and national ethical clearance from the national institution. Finally, administrative clearance to all respective hospitals we targeted before being availed of any information at the hospital level. No respondent can participate in the study without any administrative clearance. After selecting eight out of 16 regional referrals, clearance was granted by all the selected eight regional referrals hospitals. After selecting 32 out of the 47 general hospitals, administrative clearance was granted by only 21 hospitals.**

If yes, why the sampling was not conducted at the cluster level?

**Response:**

**Lines 130-141, page 8. The sampling procedure did not take a cluster sampling approach because of a shortfall in the participation of hospitals in the northern region of the country (seven general hospitals did not grant us administrative clearance). The cluster of North would have had one general hospital compared to six general hospitals in the Central, seven general hospitals in Eastern and seven in the western. There was an imbalance in terms of general hospitals. The North would not form a cluster because of the non-participation of six hospitals. Therefore, there would be a shortfall in the study population (healthcare providers) representation northern region. Furthermore, this would mean there would be one cluster with no similar characteristics in terms of healthcare providers or participating types of hospitals compared to other regions.**

**However, since there was clustering among healthcare providers in terms of cadres at the lowest level, the study adjusted for clustering in the design effect of sample size determination also adjusted for clustering during analysis using robust standard errors.**

**Comment Reviewer #2**: 
Thanks for indicating later that the sample consists mostly of same size "general hospitals" but what about the sizes of other hospitals in the sample (for example is the 1000 bed "regional referral" hospital in the sample)?

**Response:**

**Lines 143-149, page 8. Thanks for this comment. The study ensured proportionate representation among participating hospitals by using a proportionate number to size to select all other professional cadres except pharmacists, pharmacy technicians and laboratory technicians because each hospital had one cadre. The latter were purposively selected in each participating hospital.**

**Comment Reviewer #2**:  If "4 general hospitals for each" of the 8 "regional referral facility" was selected, how did the authors end up with "21 general hospitals" and with the regional distribution of the sample provided (lines 162-165)? Also, see above for what "approximately 50 general hospitals" (line 116) mean.

If "4 general hospitals for each" of the 8 "regional referral facility" was selected, how did the authors end up with "21 general hospitals"

**Response:**

**Lines 133-141, page 8. Thanks for this question. Before data collection, we selected randomly eight regional referrals hospitals. We selected 32 general hospitals out of the 47 included general hospitals. For each of the selected regional referrals, we had to select four general hospitals. Therefore for all the four regions (North, West, East and Central), we selected 32 general hospitals out of the 47. How did we end up with 21 general hospitals? After selecting the eight regional referrals, 32 general hospitals, and three tertiary PNFPs, we applied for administrative clearance in each selected health facility. All the eight regional referrals and three tertiary PNFPs granted us administrative clearance. However, for general hospitals, only 21 out of 32 granted us administrative clearance?. The ethical obligation by the Uganda National Council of Science and Technology and the parent institution (Makerere University Higher degrees ethics and research committees and the hospital research and ethics committee) can only be done once granted ethical clearance by all the three institutions. That is how 21 general hospitals appear in the stated methods.**

Also, see above for what "approximately 50 general hospitals" (line 116) mean.

**Lines 91-92, page 6. Thanks for this question, and we apologies for not being precise on using "approximately 50" general hospitals. According to the national census for health facilities carried out in 2014 and the national health facility master list, there are 47 general hospitals in Uganda.**

**Comment Reviewer #2**: 
Thanks for information about 2 "national referral" hospitals (new lines 102-105) that were excluded. Is there a 3rd one?

**Response:**

**Lines 91-92, page 6. By 2014 when a national census for health facilities was done, there were three national referrals, Mulago super specialised hospitals, Butabika national referral for mental health, and Buluba national referral for leprosy. In the latest health facility master list 2018, the Ministry of Health no longer recognises Buluba as a national referral, and it implies we are left with two national referral hospitals.**

**Comment Reviewer #2**: 
Please see above; "21 general hospitals" is still not clear to the this reviewer.

**Response:**

**Lines 144-156, page 8. Thanks for this question. Before data collection, we selected randomly eight regional referrals. Then we selected 32 of the 47 general hospitals, where for each of the selected regional referrals, we had to select four general hospitals. We selected 32 general hospitals out of the 47. How did we end up with 21 general hospitals? After selecting the 32 general hospitals, we applied for administrative clearance in each of the selected general hospitals. Only 21 general hospitals granted us the clearance to conduct the study with them. These included one in the North, six in the central, seven in the East and seven in the West. The ethical obligation to the Uganda National Council of Science and Technology and the Makerere University Higher degrees ethics and research committee, and the hospital research and ethics committee was that the researcher could only conduct data collection after all three institutions had received ethical clearance. That is how the 21 general hospitals appear stated in our methods.**

18. "Old Lines 159-218: The reviewer finds it impossible to interpret the results without clarifications for the points above."

Response :

**Lines 132-141, page 8. After clarifying the information on the number of hospitals that participated and how they were selected we believe the reviewer will agree with us on this revision that results can be easily interpreted**

**Comment Reviewer #2**: 
This reviewer still finds it impossible. Did authors analyse their data as if it was a random sample?

**Response:**

**The answer is a yes or no. We had chosen facilities randomly, but the healthcare providers like pharmacists, laboratory technicians and pharmacy technicians were chosen purposively since we only found one person per health facility. In contrast, nurses, medical officers, medical specialists, and clinical officers all were chosen by simple random sampling using sampling frames of the hospital facilities. Having a sample with a mixture of persons who were picked purposively and others randomly the answer may lie most on the no side.**

**Comment Reviewer #2**: 

20. Old "Please avoid typos and ensure completeness and transparency in the manuscript:

**Response**

**Thanks for this observation, we have corrected all typos in the manuscript**

a) please ensure that all of the acronyms (such as, AMS, in line 25), are spelled out the first time they are mentioned (it is in the Abstract but also needs to be spelled out in the body of the manuscript),

**Response**

**Lines 2, page 1 and Lines 36, page 2. Thanks for the correction, We have spelt out the word AMS twice in the abstract and once in the main section**

b) please ensure that the references are complete (such as #18, for example, please be clear about "WHO" and the location of the publisher…),

**Response**

**Lines 498-601: Thanks for the correction, and we have ensured the references are all complete.**

c) "would; reduce" (?) in line 173 or "is has been" (?) in line 257, d) please ensure that tables are self-explanatory (see, for example, Table 2, what are "AMS", "60 points" and what does the second note refer to, or Table 4, why "HCP"…)"

**Response**

**Lines 271 pages 16. Thanks for the correction, "would reduce" corrected to "minimises."**

**Lines 390 pages 27. "is has been" corrected to "has been".**

**Table 2: We reported the mean score in 12 questions on antimicrobial stewardship attitudes. This has been improved.**

**Table 4: Sorry for the HCP. It has been corrected.**

Some of the new points:

a) new line 27, do you mean "arises",

Response

**Lines 28-29: we corrected the statement.**

b) new line 33, the sentence that starts with "Antimicrobial stewardship (AMS)…" needs to be a new paragraph,

**Response**

**Lines 37: We thank you for this suggestion. We have made this correction.**

b) new line 61, "where" seems to be missing, d) "LMIC"? new line 76….

**Lines 73, pages 2: we have corrected the abbreviation**

7. PLOS authors have the option to publish the peer review history of their article ([what does this mean?](https://journals.plos.org/plosone/s/editorial-and-peer-review-process#loc-peer-review-history)). If published, this will include your full peer review and any attached files.


If you choose "no", your identity will remain anonymous but your review may still be made public.
No

**Do you want your identity to be public for this peer review?** For information about this choice, including consent withdrawal, please see our [Privacy Policy](https://www.plos.org/privacy-policy).

Reviewer #1: No

Reviewer #2: No

 No

[NOTE: If reviewer comments were submitted as an attachment file, they will be attached to this email and accessible via the submission site. Please log into your account, locate the manuscript record, and check for the action link "View Attachments". If this link does not appear, there are no attachment files.]

While revising your submission, please upload your figure files to the Preflight Analysis and Conversion Engine (PACE) digital diagnostic tool, <https://pacev2.apexcovantage.com/>. PACE helps ensure that figures meet PLOS requirements. To use PACE, you must first register as a user. Registration is free. Then, login and navigate to the UPLOAD tab, where you will find detailed instructions on how to use the tool. If you encounter any issues or have any questions when using PACE, please email PLOS at [figures@plos.org](mailto:figures@plos.org). Please note that Supporting Information files do not need this step.
