# Supplementary material for: Antimicrobial stewardship: Attitudes and practices of healthcare providers in selected health facilities in Uganda
Source: PLoS One. 2022 Feb 3;17(2):e0262993. doi: 10.1371/journal.pone.0262993 (PMC8812957; doi:10.1371/journal.pone.0262993)
Supplement: Attachment — Submitted filename: response to reviewers.docx [file pone.0262993.s007.docx]

**RESPONSE TO REVIEWER'S COMMENTS ON MANUSCRIPT, PONE-D-21-10939R2**

Dear Editor:

We appreciate the comments. We have revised our manuscript according to the editor and reviewers' comments, questions, and suggestions. We appreciate the comments and feedback since they have improved our manuscript. We wish you a happy new year.

**Yours,**

**Isaac Kimbowa**

**Corresponding author**

**RESPONSE TO REVIEWER'S COMMENTS ON MANUSCRIPT, PONE-D-21-10939R2**

Thanks for an improved manuscript. The following needs to be taken into consideration.

**Comment # 1, Reviewer 1:** 1. "Old Lines 33-50: Could authors add more information to motivate their study of AMS attitudes and practices of healthcare providers? Is this the most important aspect of reducing antibacterial resistance in Uganda? Is the paucity of information on this specific aspect? How was the National Action Plan on Antimicrobial Resistance drafted; bottom-up or top-to-bottom? When drafted? Is it implemented? If yes, how and when (thanks, some information is provided in the Discussion section (lines 227-229) but it needs to be clear from the start) …" Thanks for revisions;

**Response**

**We thank you for the valuable comments**

**Comment #2, Reviewer 1:** the Introduction section motivates the study well. However, it is now more than 3 pages long. Please consider shortening it without losing any of its content.

**Response**

**Lines 26-88, Thanks for the comment. The introduction section in the revised manuscript is currently two pages.**

**Comment #3, Reviewer 1:** "Old Lines 53-57: Could authors provide more information about the characteristics of these different hospitals, such as their bed size, teaching status, location characteristics (urban or rural…), their regional distribution etc? Are there any for-profit hospitals in Uganda?" Thanks for providing more information.

**Response**

**Noted**

**Comment #4 , Reviewer 1**: This section needs to be shortened and, please, just provide information that is relevant to motivate the sampling strategy.

**Response**

**Thanks for the comment. We have provided relevant information that motivates the sampling strategy in the methods section of the revised manuscripts**

**Comment #5, Reviewer 1:** Specific questions: (a) Thanks for information about 2 (or is it 3?) "national referral" hospitals (new lines 102-105) that were excluded. As a result, (and names are not required but) what % of the public health hospitals were excluded?

**Response**

a) **In Uganda, there are two national referrals, 16 regional referrals and 47 general hospitals. In total, there are 65 public hospitals in the country. In our study, we included 63 out of 65 (96.9%) public hospitals. The two hospitals which were not included were both national referral hospitals………... Lines 94-96, pages 6**

**Comment #6, Reviewer 1:** Specific questions (b) “two million” (line 107)?

**Response**

b) **According to the Uganda Master list of health facilities of 2018, page 7, each health facility has a designated population size that it is meant to serve: a national referral hospital (10,000,000 people), each regional referral hospital (2,000,000 people), general hospitals (500,000 people), and Health Center IV (100,000), Health Center III (20000), Health Center II (5000), and Health Center I (not defined).**

**In the current revised manuscript, we have removed this information……. Lines 98-100…pages 6,**

**Comment #7, Reviewer 1:** Specific questions (c) Which region is this; (again, we do not need names and the detail but) why "five regional referral hospitals" followed with 4 names (lines 108-109)?

**Response**

c) **In the initial submission, we had accidentally omitted to list the name of the fifth regional referral in the central region. The names of all the five regional referrals in the central region are; Masaka, Mubende, Kayunga, Old Mulago, Naguru.**

**However, from the subsequent reviews, reviewers recommended we omit the names of the health facilities. Therefore, this information has been removed from the current revised manuscript.……… Lines 99-104, page 6.**

**Comment #8, Reviewer 1:** Specific questions (d) What does "approximately 50 general hospitals" (line 116) mean or are there 41 (lines 115-124) general hospitals?

**Response**

d) **We appreciate this comment. We have 47 general hospitals as provided in the master list of health facilities 2018. ……………. Lines 96, pages 6.**

**Comment #9, Reviewer 1:** Specific questions (e) Please be clear, do these hospitals have about 10 beds each (lines 123-124) or 100 beds?

**Response**

e) **Sorry about the typo. We meant all general hospitals have 100 beds………. lines 100, page 6**

**Comment #10, Reviewer 1:** Specific questions (f) If there are "44" private not-for-profit (PNFP) hospitals and if "3 out of 4" that are considered as "regional referral centres" were included in this study, what % of PNFPs was excluded from this study?

**Response**

**a) In our study, we only considered tertiary level PNFPs, of which there are four in the country. We selected three tertiary PNFPs out of four.……………… lines 143, pages 8.**

**Comment #11, Reviewer 1:** 3. "Old Lines 53-57: Could authors explain their reasons for their focus on hospitals only? Are the AMS programmes focus at hospitals?" Thanks for explanations.

**Response**

**Noted**

**Comment #12, Reviewer 1:** 3 "Old Lines 53-57: In the healthcare system, are hospitals the only healthcare institution type where antibacterial misuse is observed?

**Response**

**Thanks for your comment. Its true antibacterial misuse occurs at all levels of the healthcare system, and it is not limited to regional referrals, general hospitals, and PNFPs. Information on antibacterial misuse has been published widely studied in Uganda by several authors such as Mukonzo et al., 2013; Ssekikubo *et al.,* 2017; Ocan et al., 2014,2015. The authors have included issues on misuse in the introduction and rationale for selecting health facilities….. Page 6, lines 105-106.**

**Comment #13 , Reviewer 1:** 5. "Old Lines 69-71**:** Thanks for the information. This publication requires that at least 3 "sectors" to be identified as a 1st step. So, as before, which are the important "sectors" for the subject matter of this study, only hospitals?"

**Response**

**Our study focused on healthcare workers in hospitals due to the existence of formal antimicrobial stewardship programmes intended to guide antimicrobial use. Our study sought to establish the attitudes and practices of healthcare providers on antimicrobial stewardship programmes in their hospital…………. lines 43-47; page 4**

**Comment #14, Reviewer 1:** 5. "Old Lines 69-71: This section in its new format now indicates that information about total number of observational units were available to authors.

**Response**

**Noted**

**Comment #15: , Reviewer 1:** 5. "Old Lines 69-71: Was this information also available at the hospital level? If yes, why the sampling was not conducted at the cluster level?

**Response**

**No. The information was not available to the authors so cluster sampling could not be done……… lines 135-147, pages 8.**

**Comment #16, Reviewer 1:** 5. "Old Lines 69-71: Thanks for indicating later that the sample consists mostly of same size "general hospitals" but what about the sizes of other hospitals in the sample (for example is the 1000 bed "regional referral" hospital in the sample)?

**Response**

**Thanks for this comment.**

**We included general hospitals with a 100 bed capacity. We also included regional referral and PNFPs hospitals whose bed capacity ranged between 250 to 600 beds.……….……. shown by Lines 147-151, page 8.**

**Comment #17, Reviewer 1:** 6. "Old Lines 71-74: Thanks for the information. However, it is unclear to the readership how the increase from 4 to 8 regional facilities should be interpreted?

**Response**

**Thanks for this question.**

**This comment is noted. However, looking through all old versions of the manuscripts, we did not mention four regional referrals. The old lines 71 to 74 mentioned by the reviewer are in the introduction section. Our study selected eight regional referrals as indicated in the methods sections ……….Lines 133 to 145, pages 8.**

**Comment #18** **, Reviewer 1:** 6. "Old Lines 71-74: The recommendation of "4" is per sector in the publication. Again, are there other "sectors" that are of importance for the subject matter?" Thanks for revisions.

**Response**

**Noted**

**Comment #19** **, Reviewer 1:** 6. "Old Lines 71-74: If "4 general hospitals for each" of the 8 "regional referral facility" was selected, how did the authors end up with "21 general hospitals" and with the regional distribution of the sample provided (lines 162-165)?

**Response**

**True, four general hospitals were selected for each of eight regional referral hospitals, thus giving us 32 general hospitals that could potentially be included in the study. However, when all the 32 were contacted for enrollment (administrative clearance), only 21 consented and were therefore enrolled in the study. …….Page 8, lines 133-145.**

**Comment #20 , Reviewer 1:** 6. "Old Lines 71-74: Also, see above for what "approximately 50 general hospitals" (line 116) mean.

**Response**

**This was an error in stating the number of general hospitals. However, in the national health facility master list, there are 47 general hospitals in Uganda. This has been corrected in the new manuscript ---------------------- Lines 89-90, page 6.**

**Comment #21** **, Reviewer 1:** 9. "Old Lines 82-86: Please be clear if this means that none of the 3 "national referral hospitals" (lines 54-55) were selected into the sample. If so, please provide the rationale."

**Response**

**None of the national referrals was included in this study. We could not obtain administrative clearance from them.**

**Comment #22** **, Reviewer 1:** 9. "Old Lines 82-86: Thanks for information about 2 "national referral" hospitals (new lines 102-105) that were excluded. Is there a 3rd one?

**Response**

**Noted. Our study did not include any national referral hospitals.……… Pages 6; lines 91-92.**

**Comment #23** **, Reviewer 1:** 12. "Old Lines 83-85: Please be clear how 3 "general hospitals" in each to the 4 regions add up to "21 general hospitals" (lines 72-73 or line 300) and, therefore, to "32 health facilities" overall (lines 73-74 or Abstract, line 15) for the study?" Please see above;

**Response**

**Thank you for the comment. Information on the number of hospitals included in the study has been clarified in the revised manuscript.…….. Lines 137-145, page 8.**

**Comment #24, Reviewer 1:** 12. "Old Lines 83-85: "21 general hospitals" is still not clear to the this reviewer.

**Response**

**This has been clarified in the revised manuscript.………………. lines 137-145, page 8.**

**Comment#25, Reviewer 1:** 18. "Old Lines 159-218: The reviewer finds it impossible to interpret the results without clarifications for the points above."

**Response**

**This is noted. We have improved the description of the methods section. It should now be easier to follow and will make interpretation of our manuscript easier**

**Comment #26, Reviewer 1:** This reviewer still finds it impossible. Did authors analyse their data as if it was a random sample?

**Response**

**In the analysis of our data , we adjusted for clustering of the study units**

**Comment #27, Reviewer 1**: 20. Old "Please avoid typos and ensure completeness and transparency in the manuscript:

**Response**

**Thanks for this observation. We have corrected all typos in the manuscript**

**Comment #28, Reviewer 1:** a) please ensure that all of the acronyms (such as, AMS, in line 25), are spelled out the first time they are mentioned (it is in the Abstract but also needs to be spelled out in the body of the manuscript),

**Response**

**Lines 2, page 1 and Lines 36, page 2. Thanks for the correction, We have spelt out the word AMS in the abstract and introduction.**

**Comment #29, Reviewer 1:** b) please ensure that the references are complete (such as #18, for example, please be clear about "WHO" and the location of the publisher…),

**Response**

**Lines 498-601: Thanks for the correction, and we have ensured the references are all complete.**

**Comment # 30, Reviewer 1:** c) "would; reduce" (?) in line 173 or "is has been" (?) in line 257,

**Response**

**Lines 293 pages 16. Thanks for the correction, "would reduce" corrected to "minimises."**

**Comment # 31, Reviewer 1:** d) please ensure that tables are self-explanatory (see, for example, Table 2, what are "AMS", "60 points" and what does the second note refer to, or Table 4, why "HCP"…)"

**Response**

**Table 2: We reported the mean and standard deviation of AMS attitude scores of each profession in 12 questions on antimicrobial stewardship attitudes. This has been improved…………………. pages 18**

**Comment#32, Reviewer 1:** Some of the new points: a) new line 27, do you mean "arises",

**Response**

**Thanks for the comment. We corrected the statement. …………..Lines 27, pages 3.**

**Comment#33, Reviewer 1:** b) new line 33, the sentence that starts with "Antimicrobial stewardship (AMS)…" needs to be a new paragraph,

**Response**

**Thanks for this comment. We have corrected it……………. Lines 35, page 3.**

**Comments #34, Reviewer 1:** c) new line 61, "where" seems to be missing,

**Response**

**We thank you for this suggestion. We have made this correction……. on Lines 44, page 3.**

**Comments #35, Reviewer 1:** d) "LMIC"? new line 76….

**Response**

**We have corrected the abbreviation………. Lines 73, pages 5.**

-----

This revised version is significantly improved from prior. I recommend some additional minor changes prior to publication, which I have noted below.

**Comment # 36, Reviewer 2:** Title and Abstract: Unchanged from prior, no changes recommended.

**Response**

**Thanks for the comment , we appreciate it**

**Comment # 37, Reviewer 2:** Introduction: The authors have largely re-written the introduction, and it is well-written. No further changes recommended.

**Response**

**Thanks for the valuable comments, and we appreciate your time.**

**Comment # 38, Reviewer 2:** Methods and Results: Largely re-written.

**Response**

**We appreciate your comment**

**Comment # 39, Reviewer 2:** There is excessive detail on the number of hospitals in each region which is somewhat distracting to the reader.

**Response**

**Lines 99-107 on page 6; We have improved the clarity of writing in this section, deleted names of hospitals and limited ourselves to the number of targeted hospitals and the reasons for their selection.**

**Comment # 40, Reviewer 2:** The authors still have not described how recruitment/invitation to participate was carried out at each facility to reach the enrollment targets, and whether any compensation/incentive was provided.

**Response**

**We have added more information on how the study participants were enrolled. recruitment. Lines 197-205, page 11.**

**Comment # 41, Reviewer 2:** In my prior comments, I suggested categorising the AMS attitude scores as 'high', 'average', and 'low'. Re-reading the manuscript now, I think 'average' is not the right label, as this has statistical meaning as well. Consider using 'moderate' or 'fair' to label this category instead of 'average'.

**Response**

**Response: The authors want to thank the review and have agreed to use "fair" instead of "average", as reflected in this manuscript's sections.**

**Comment # 42, Reviewer 2:** Results of statistical models have been re-written and are more appropriate.

**Thanks for this comment. We appreciate it**

**Comment # 43, Reviewer 2:** In Line 348, I recommend changing the start of the sentence to 'In multivariable models adjusting for possible confounders, AMS practice scores …' This is to clarify that you are referring to your model results.

**Response**

**Thanks for the correction. We have corrected it as suggested. Lines 328-323, page 23-24.**

**Comment # 44, Reviewer 2:** Also include the AOR and its associated confidence interval in this sentence, not just the P-value for the association.

**Response**

**We have made this correction as stated above ……………lines 327-329 pages 24**

**Comment # 45, Reviewer 2:** Discussion and Conclusions: Largely re-written.

**Response**

**We thank you for this comment.**

**Comment # 46, Reviewer 2:** In line 364 I recommend avoiding the phrase 'will never', because this is unlikely to be the case.

**Response**

**Thanks for the correction. We have improved the clarity of the statement………….. Lines 340, pages 25,**

**Comment # 47, Reviewer 2:** Consider rephrasing as 'are unlikely to'.

**Response**

**Thanks for the correction. We have improved the clarity of the statement ……. Lines 333-334, pages 25**

**Comment # 48, Reviewer 2:** In addition, there is only one limitation addressed – about potential bias in the interview.

**Response**

**Thank you for this concern. We added other potential biases (selection bias, non-response) that could have arisen from the study………………….. Lines 421-428 pages 29**

**Comment # 49, Reviewer 2:** I think additional limitations should be added concerning the lack of data gathered from trainees (though part-time employees, they do prescribe a significant proportion of antibiotics and have their own AMS attitudes and practices, which were not measured here),

**Response**

**Lines 428-429, page 29. We have included the limitation of part-time and intern healthcare providers.**

**Lines 432-433, Pages 29. We have added a limitation on the uncertainty surrounding the cause and effect of AMS attitudes and practices.**

**Comment # 50, Reviewer 2:** uncertainty around the cause and effect of AMS attitudes and practices, and potential lack of generalizability of your results to non-Ugandan or non-East African settings.

**Lines 433-435, Pages 29: Under the limitation section, we have included the lack of generalizability to the non-East African setting.**

**Our study did not set out to confirm the causal relationship between antimicrobial stewardship attitudes and practices. However, we reported on the associations between the attitudes practices and the healthcare providers of the hospitals.**

-----
